# Secretory carrier membrane protein 3 modulates nuclear ERK1/2 signaling and drug response in triple-negative breast cancer

**DOI:** 10.3389/fonc.2026.1854966

**Published:** 2026-09-14

**Authors:** Beatriz M. Morales-Cabán, Millenia Jackson-González, Fernando A. Acosta-Pérez, Bryan S. Ortiz-Torres, Ivette J. Suárez-Arroyo

**Affiliations:** 1 Department of Biochemistry, School of Medicine, Universidad Central del Caribe, Bayamón, PR, United States; 2 Department of Industrial and Systems Engineering, University of Wisconsin-, Madison, WI, United States

**Keywords:** ERK1/2, MK-8353, RAS/RAF, SCAMP3, triple-negative breast cancer

## Abstract

**Introduction:**

Triple-negative breast cancer (TNBC) frequently displays hyperactive mitogen-activated protein kinase (MAPK)/extracellular signal-regulated kinases 1/2 (ERK1/2) signaling but lacks tractable targets to sustain durable pathway inhibition. Identifying modulators that shape ERK1/2 output and influence responses to ERK1/2 inhibitors is therefore needed. The endosomal trafficking protein secretory carrier membrane protein 3 (SCAMP3) is a positive regulator of epidermal growth factor receptor (EGFR) signaling and ERK1/2-dependent phosphoproteomics programs in TNBC. However, the mechanism by which SCAMP3 modulates ERK1/2 signaling and targeted drug response remains unclear.

**Methods:**

Here, we combined SCAMP3 knockout with pharmacological ERK1/2 inhibition to evaluate SCAMP3's contributions to MAPK signaling, ERK1/2 distribution, cell-cycle control, apoptosis, and *in vivo* drug response.

**Results:**

We found that SCAMP3 expression is upregulated in TNBC models harboring intrinsic MAPK-activating alterations compared with MAPK wild-type breast cancer cells. SCAMP3 knockout reduced RAF-MEK-ERK1/2 signaling, decreased nuclear accumulation of active ERK1/2, arrested the cell cycle, and promoted apoptotic responses. Furthermore, SCAMP3 knockout increased the inhibitory response to the ERK1/2-targeted inhibitor MK-8353 in xenograft models of MAPK wild-type TNBC.

**Conclusion:**

This study identifies SCAMP3 as an important modulator of ERK1/2 signaling and supports further investigation of SCAMP3 as a potential therapeutic co-target to improve ERK1/2-directed therapies.

## 1 Introduction

Triple-negative breast cancer (TNBC) is characterized by high proliferative rates, early brain metastasis, frequent relapse, and poor overall survival. Defined by the absence of estrogen receptor (ER) and progesterone receptor (PR) expression and by the absence of human epidermal growth factor receptor 2 (HER2) overexpression, TNBC is unresponsive to targeted therapies commonly employed in other breast cancer subtypes (1–3). Recent advances, including immune checkpoint inhibitors, PARP inhibitors, and antibody-drug conjugates, have improved outcomes for selected biomarker-defined subsets. However, these advances are limited by toxicity, rapid resistance emergence, and limited durability of response (4, 5). These limitations underscore the need to define key signaling nodes that drive TNBC progression and resistance, thereby improving the design of pathway-targeted strategies. The RAS/RAF/MEK1/2/ERK1/2 MAPK cascade integrates receptor-derived and oncogenic inputs to control proliferation, survival, and stress adaptation (6, 7). Following growth factor stimulation, ERK1/2 is activated at the plasma membrane and in endomembranes. It then translocates to the nucleus, where it phosphorylates transcription factors that drive cell cycle progression, apoptosis resistance, and feedback regulation of upstream kinases (8–10). In TNBC, persistent MAPK activity predominantly results from dysregulated receptor tyrosine kinase (RTK) signaling, particularly epidermal growth factor receptor (EGFR), which is overexpressed in approximately 40-60% of cases and is associated with poor clinicopathologic features (11–13). Aberrant EGFR trafficking sustains multiple oncogenic cascades, including PI3K/AKT, mTOR, Jak/STAT3, and MAPK/ERK1/2 pathways, thereby promoting invasion, metastasis, and chemoresistance (12–15). Although RAS/RAF mutations are less frequent in TNBC (<5%), activating mutations such as HRAS G12D, KRAS G13D, BRAF G464V, and NRAS overexpression further amplify ERK1/2 signaling and have been linked to aggressive behavior and therapeutic escape (16–19). Together, these receptor-driven and mutational mechanisms converge on ERK1/2 as a critical determinant of TNBC progression.

Upstream mutations or RTK levels do not solely determine MAPK output; they are also influenced by endocytic trafficking and scaffold networks that govern nuclear signaling complexes (20, 21). Adaptor and scaffold proteins such as CBL, GRB2, SHC1, and endosomal MAPK scaffolds couple RTK internalization to ERK1/2 signaling (22–24). Within this network, Secretory Carrier Membrane Protein 3 (SCAMP3), a tetraspanning membrane protein enriched in endosomes and the trans-Golgi network, has emerged as a key regulator of EGFR trafficking and signal routing (22–25). SCAMP3 is upregulated in several malignancies, including hepatocellular carcinoma, glioma, melanoma, pancreatic, and breast cancer, where high expression correlates with aggressive features, poor prognosis, and therapy resistance (26–33). We and others have demonstrated that SCAMP3 regulates EGFR stability and endosomal routing, supports mitogenic and survival signaling, and promotes proliferation, migration, and invasion (25, 26, 28, 34). More recently, phosphoproteomics profiling demonstrated that SCAMP3 maintains ERK1/2-dependent phosphorylation programs and couples ERK1/2 activity to mTORC1 signaling and autophagy, positioning SCAMP3 as an organizer of ERK1/2-driven stress adaptation (29). However, how SCAMP3 modulates the integrity of the RAF-MEK-ERK1/2 cascade, controls nuclear engagement of ERK1/2, and influences responses to ERK1/2 inhibition in distinct TNBC genotypes remains poorly defined.

Direct pharmacological targeting of ERK1/2 has emerged as a strategy to overcome resistance to upstream RAF or MEK inhibition (35). MK-8353 is an orally bioavailable ATP-competitive ERK1/2 inhibitor that has shown target engagement, MAPK pathway suppression, and antitumor activity in preclinical models and early-phase clinical trials in advanced solid tumors. Nevertheless, adaptive feedback and pathway reactivation limit the efficacy of ERK1/2 inhibition (35–37). Thus, it is crucial to identify critical modulators of ERK1/2 output and drug response.

In this study, we identified a significant association between SCAMP3 expression and MAPK pathway activation in TNBC. We further showed that genetic targeting of SCAMP3 significantly inhibits tumor growth and enhances the antitumor efficacy of MK-8353. These findings reveal how SCAMP3 contributes to TNBC growth and provide additional preclinical evidence for its potential as a therapeutic co-target for monotherapy and combination regimens.

## 2 Materials and methods

### 2.1 Cell culture and reagents

SUM-149, SUM-159, and SUM-190 cells were obtained from BioIVT (Westbury, NY, USA). MDA-MB-468 and MCF-10A cells were obtained from the American Type Culture Collection (ATCC, Manassas, VA, USA) and authenticated by the distributor. Mycoplasma testing was performed using the Mycoplasma Detection Kit (Nordic BioSite AB, Taby, Sweden). SUM-149 and SUM-159 cells were cultured in Ham’s F-12 Nutrient Mixture supplemented with 10% fetal bovine serum (FBS; Corning, Corning, NY, USA). SUM-190 cells were cultured in Ham’s F-12 supplemented with 2% FBS, 1 g/L bovine serum albumin (BSA), 100X insulin-transferrin-sodium (ITS), and 6.6 ng/mL triiodo-L-thyronine sodium salt (T3) (Sigma-Aldrich, St. Louis, MO, USA). MDA-MB-468 cells were cultured in Dulbecco’s Modified Eagle Medium (DMEM) containing 10% FBS. MCF-10A cells were maintained in a 1:1 mixture of DMEM and Ham’s F-12 supplemented with 10% horse serum, 20 ng/mL epidermal growth factor (EGF), 100 ng/mL cholera toxin, 1 μg/mL hydrocortisone, and 10 μg/mL insulin (Sigma-Aldrich). All cultures were maintained at 37 °C with 5% CO_2_. The ERK1/2 inhibitor MK-8353 was purchased from MedChemExpress (Monmouth Junction, NJ, USA).

### 2.2 Lentiviral transduction

SCAMP3-deficient (SC3KO) SUM-149 and MDA-MB-468 cell lines were generated previously by CRISPR/Cas9-mediated gene editing followed by clonal isolation and were validated at both the genomic and protein levels, as previously described in (26). These previously characterized clonal cell lines were used throughout the present study. To generate SCAMP3-deficient SUM-190 cells, CRISPR/Cas9 was delivered via lentiviral vectors as previously described (26). Viral supernatant was collected 36 h after co-transfection of the sgRNA plasmid (lentiCRISPRv2.0 plasmid #52961; Addgene, Watertown, MA, USA) together with the packaging plasmids pCMV-dR8.2 and pMD2.G (Addgene) into HEK-293T cells using FuGENE HD transfection reagent (Promega, Madison, WI, USA). After transduction, cells were selected with 1 µg/mL puromycin (Sigma-Aldrich).

### 2.3 Cell viability assay

Cells (2.0-6.0 x 10^4^/well) were seeded in 48-well plates and treated with vehicle (DMSO) or serial concentrations of MK-8353 for 24 h. Cells were fixed with cold methanol and stained with 0.4 mg/mL propidium iodide (PI; Sigma-Aldrich). Fluorescence was measured at 620 nm using a GloMax^®^ Microplate Reader (Promega).

### 2.4 Protein extraction and Western blot assay

Whole-protein extracts and immunoblots were prepared using established methods (26, 29). Nuclear fractions were prepared using the Nuclear Extract Kit (Active Motif, Carlsbad, CA, USA). The following primary antibodies were used. From Cell Signaling Technology (Danvers, MA, USA): B-Raf (E3T5C, #77622), phospho-B-Raf (Ser445, #2696), ERK1/2 (#9102), phospho-ERK1/2 (Thr202/Tyr204, #4370), Cyclin B1 (D5C10 XP^®^, #12231), Cyclin E1 (D7T3U, #20808), Cyclin D1 (#2922S), Caspase-3 (D3R6Y, #14220), Caspase-9 (C9, #9508), PARP-1 (#9542), MLKL (D2I6N, #14993), phospho-MLKL (Ser358, D6H3V, #91689), β-Actin (8H10D10, #3700), β-tubulin (#86298). Primary antibodies from Santa Cruz Biotechnology (Dallas, TX, USA) included Importin-7 (sc-365231), MEK-1/2 (9G3, sc-81504), p-MEK-1/2 (S218/222; S222/226, 7E10, sc-81503), ELK-1 (E-5, sc-365876), p-ELK-1 (S358 B-4, sc-8406), c-Fos (E-8, sc-166940), p-c-Fos (S374, 34E4), Ran (A-7, sc-271376), and Lamin A/C (E-1, sc-376248). SCAMP3 antibody (#PA5-21428) was from Thermo Fisher Scientific (Waltham, MA, USA). Appropriate HRP-conjugated or fluorophore-conjugated secondary antibodies were applied for 1 h. Immunoreactive bands were visualized using either the ChemiDoc imaging system (Bio-Rad, Hercules, CA, USA) or an Odyssey DLx Imager (LI-COR Biosciences, Lincoln, NE, USA). Band intensities were quantified using ImageJ software (v1.54p).

### 2.5 Cell cycle assay

5.0 x 10^5^/well cells were seeded in 6-well plates and treated for 24 h with EGF (10 ng/mL), vehicle (DMSO), or MK-8353 (1 μM). Serum-starved cells served as negative controls. Cells were trypsinized using 0.05% trypsin-EDTA (Thermo Fisher Scientific, Waltham, MA, USA), washed with cold PBS, and fixed in 70% ethanol overnight. After staining with Guava Cell Cycle Reagent (Luminex, Austin, TX, USA), samples were analyzed using a Guava easyCyte 8 Flow Cytometer (Luminex) and data processed using GuavaSoft v3.1.1.

### 2.6 Flow cytometry analysis of apoptosis

Apoptosis was measured using Annexin V-FITC and PI (Nexcelom Bioscience, Lawrence, MA, USA). Cells (1.0 × 10^6^) were treated with vehicle or MK-8353 (2 μM) for 24 h, stained for 15 min in the dark, and analyzed using the Cellometer™ Spectrum (Nexcelom Bioscience). Data were quantified using FCS Express (Dotmatics, San Diego, CA, USA). Dot plots were displayed as Annexin V FITC (x axis) versus PI (y axis), and quadrants defined four populations: live (Annexin V^–^/PI^–^, lower left), early apoptotic (Annexin V^+^/PI^–^, lower right), late apoptotic/secondary necrotic (Annexin V^+^/PI^+^, upper right), and debris (upper left quadrant). The percentage of apoptotic cells reported in the figures corresponds to the early apoptotic population (Annexin V^+^/PI^–^), as determined using FCS Express. Late apoptotic/necrotic cells (Annexin V^+^/PI^+^) are presented in the figures under the label “Necrotic”.

### 2.7 Immunofluorescence

Cells (3.0 x 10^4^) were seeded on coverslips, serum-starved for 18 h, and treated with EGF (10 ng/mL) or MK-8353 (8 μM) for 2 h, then were fixed with 4% paraformaldehyde, permeabilized with 0.5% Triton X-100, and blocked with BSA (1 mg/mL). Cells were incubated with 1:400 diluted anti-p-ERK1/2 (Thr202/Tyr204) (Santa Cruz Biotechnology, Dallas, TX, USA) and anti-SCAMP3 (Thermo Fisher Scientific, Waltham, MA, USA). Secondary antibodies (Alexa Fluor 488 and 594) were purchased from Cell Signaling Technology (Denver, MA, USA). Nuclei were stained with DAPI. Images were captured using a Nikon Eclipse Si microscope (Nikon, Tokyo, Japan) and quantified using Nikon NIS-Elements AR v4.50. Percentage co-localization was calculated by dividing the co-localization area by the total cell area and multiplying by 100. For fluorescence intensity quantification, individual cells were identified by segmentation based on the DAPI channel using NIS-Elements AR v4.50. The mean fluorescence intensity per cell was measured for each marker, normalized to the mean fluorescence intensity per cell of untreated wild-type (NT WT) cells, and then multiplied by 100 to obtain relative fluorescence intensity (%). Colocalization between SCAMP3 and p-ERK1/2 was quantified using an object-based, segmentation-dependent workflow. Images were thresholded to generate binary masks for each channel, and colocalized objects were defined as the logical intersection of the two masks. The area and mean fluorescence intensity of the colocalized objects were measured exclusively within the intersection layer. Percentage colocalization was calculated by dividing the colocalized area by the total cell area and multiplying by 100, as previously described in (26).

### 2.8 Ethics and *in vivo* studies

All animal procedures were approved by the institutional Animal Care and Use Committee (IACUC) of Universidad Central del Caribe and were conducted in accordance with institutional and applicable national guidelines for the care and use of laboratory animals. Female SCID mice (Charles River Laboratories, Wilmington, MA, USA) were used in two orthotopic xenograft models with 1.5x10^6^ SUM-149 WT or SC3KO cells suspended in Matrigel (BD Biosciences, San José, CA, USA). Once tumors reached ~100 mm³, mice were treated with vehicle or MK-8353 (30 mg/kg twice daily or bis in die (BID)) by oral gavage (10 mice/group). Body weight and tumor volume were monitored twice weekly for 27 days. In the MDA-MB-468 study, mice were implanted with 2.0×10^6^ MDA-MB-468 WT and SC3KO cells suspended in Matrigel. Body weight and tumor volume were monitored weekly. Tumor volume was calculated as (width^2^ x length)/2 using caliper measurements. At the experimental endpoint, mice were anesthetized with 4% isoflurane and euthanized via cervical dislocation. Tumors were collected for downstream analyses.

### 2.9 Statistical analysis

IC_50_ values were calculated using nonlinear regression (log inhibitor vs normalized response). *In vitro* significance was determined by one-way ANOVA with Dunnett’s or two-way ANOVA with Tukey’s multiple comparisons from a minimum of three independent experiments using GraphPad Prism v10.4.2 (GraphPad Software, San Diego, CA, USA). *In vivo* analyses were performed using SPSS v28.0.1.0 (IBM Corp., Armonk, NY, USA) and RStudio (Boston, MA, USA). Repeated measures ANOVA compared four study groups at eight time points, with Mauchly’s test for sphericity, Shapiro-Wilk for normality, and Levene’s test for homogeneity. When variances were unequal, Dunnett T3 *post hoc* was used. A p-value ≤ 0.05 was considered significant. Individual data points represent independent biological replicates, and data are presented as mean ± SD. For cell viability assays, tumor growth curves, and mouse body-weight measurements, data are presented as mean ± SEM.

### 2.10 Illustrations

Schematics were created using BioRender (Toronto, ON, Canada) under (#PN298YNXY7).

## 3 Results

### 3.1 TNBC cells with MAPK-activating alterations express high levels of SCAMP3

To examine the relationship between SCAMP3 expression and MAPK pathway status, we analyzed SCAMP3 levels in a panel of breast cancer cell lines with MAPK signaling-activating alterations, including TNBC models such as SUM-159 (HRAS G12D), MDA-MB-231 (KRAS G13D and BRAF G464V), and MDA-MB-468 (NRAS overexpression). Wild-type RAS/RAF SUM-149 (TNBC) and SUM-190 (HER2^+^) were used as controls (16, 17, 19). As shown in **Figure 1A**, SCAMP3 abundance was significantly higher in TNBC cell lines harboring MAPK pathway-activating alterations than in the RAS/RAF wild-type TNBC line, SUM-149. In SUM-159 cells, SCAMP3 was detected as a doublet (~34–37 kDa), similar to previous reports, although the basis for this pattern remains unclear (38, 39).

**Figure 1:**
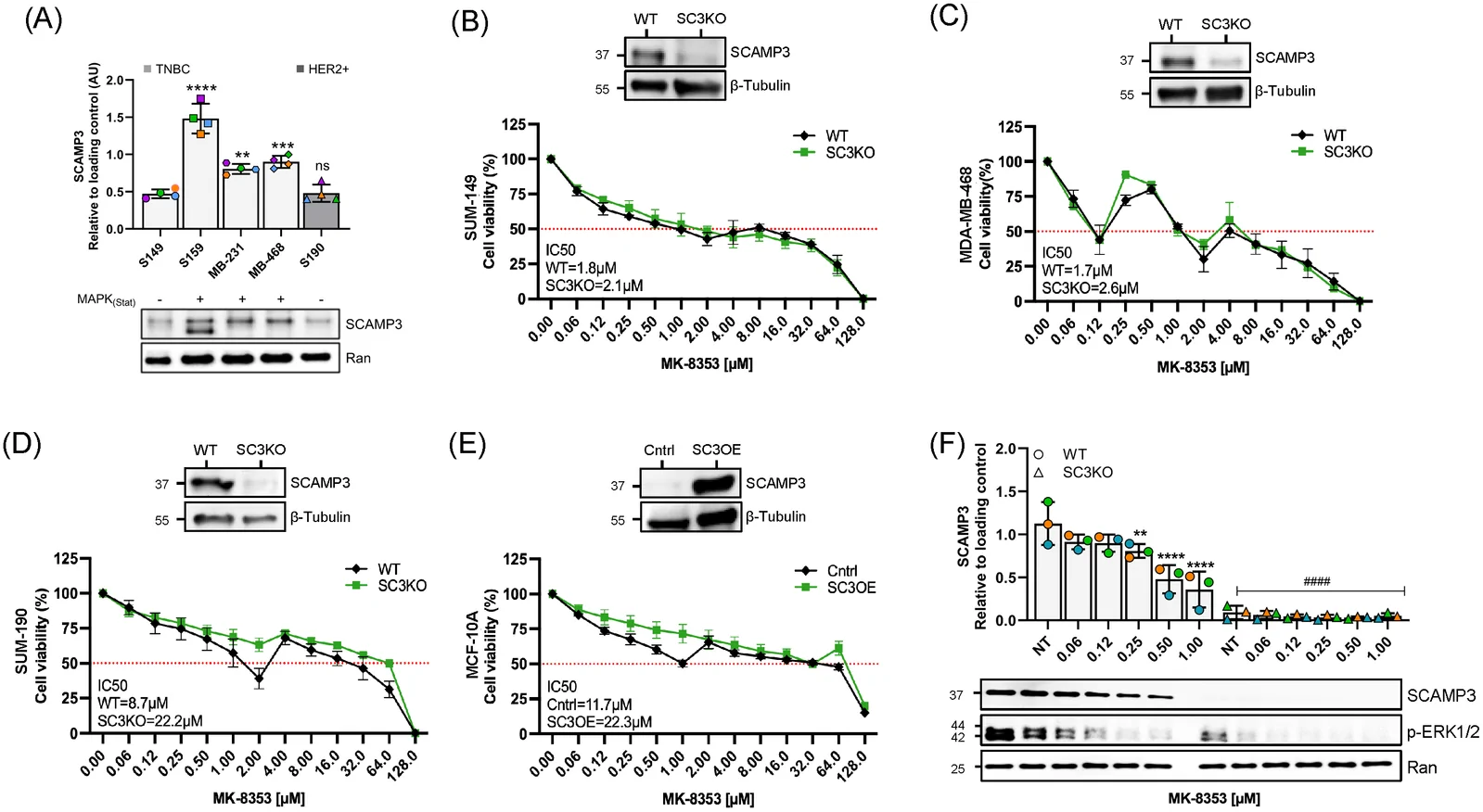
SCAMP3 expression and response to ERK1/2 inhibition in breast cancer cells. **(A)** Immunoblot and densitometric quantification of SCAMP3 protein in breast cancer cell lines: SUM-149 (TNBC; RAS/RAF_Wild-Type_), SUM-159 (TNBC; HRAS_Mutant_), MDA-MB-231 (TNBC; KRAS/BRAF_Mutant_), MDA-MB-468 (TNBC; NRAS_overexpressed_), and SUM-190 (HER2+; MAPK_WT_). Ran was used as a loading control. Data were analyzed using one-way ANOVA with Dunnett´s multiple comparison test and are reported as mean ± SD (n = 4 independent experiments). ns = not significant; **P < 0.01, ***P < 0.001, ****P < 0.0001 vs. SUM-149 expression. Cell viability curves of **(B)** SUM-149, **(C)** MDA-MB-468, and **(D)** SUM-190 WT and SCAMP3 knockout (SC3KO) cells treated with increasing concentrations of MK-8353 for 24 h. **(E)** Cell viability curves of MCF-10A control (Cntrl) and SCAMP3-overexpressing (SC3OE) cells following 24 h treatment with MK-8353. **(F)** Immunoblot and densitometric analysis of p-ERK1/2 and SCAMP3 in SUM-149 WT and SC3KO cells after 24 h exposure to the indicated concentrations of MK-8353. Ran was used as a loading control. Data were analyzed using two-way ANOVA with Tukey´s multiple comparison test. Individual data points represent independent biological replicates, and data are reported as mean ± SD. For cell viability assays, data are presented as mean ± SEM. (n = 3). **P < 0.01, ****P < 0.0001 vs. untreated (NT) of the same genotype; ^####^P < 0.0001 for WT vs. SC3KO at the indicated concentration.

To determine whether SCAMP3 loss affects the response to ERK1/2 inhibition, we used SCAMP3 knockout (SC3KO) cells previously generated and characterized in our laboratory, as described in the Materials and Methods section. Wild-type (WT) and SC3KO cells were treated with increasing concentrations (0-128 µM) of the ERK1/2 inhibitor MK-8353 for 24 h (26). SC3KO slightly increased IC_50_s in SUM-149 (1.8 µM to 2.1 µM) and MDA-MB-468 cells (1.7 µM to 2.6 µM) (**Figures 1B, C**). In contrast, SUM-190 SC3KO cells exhibited a marked increase in IC_50_ (22.2 µM) compared with their WT counterparts (8.7 µM) (**Figure 1D**), suggesting reduced sensitivity to MK-8353 following SCAMP3 loss in HER2^+^ cells. We previously generated a SCAMP3 overexpression model (SC3OE) using non-tumorigenic MCF-10A cells (26). As observed in **Figure 1E**, SC3OE cells exhibited resistance to MK-8353. Consistent with our previous publications, SC3KO showed reduced p-ERK1/2 in SUM-149 cells (**Figure 1F**). One possible explanation for the modest differences in IC_50S_ observed in TNBC cells is the reduction of SCAMP3 expression and p-ERK1/2 produced by MK-8353 shown in **Figure 1F**. These results suggest that the expression of SCAMP3 does not determine the cell viability response to MK-8353.

### 3.2 SCAMP3 loss regulates ERK1/2-dependent cell cycle control in TNBC

To determine whether SCAMP3 affects ERK1/2-driven cell cycle progression, parental SUM-149 and MDA-MB-468 cells and their SC3KO counterparts were treated for 24 h with serum-free medium (SFM), EGF, vehicle, or MK-8353. SFM and EGF were included as functional controls to verify cell cycle arrest and mitogenic responsiveness, respectively. In SUM-149 cells, SC3KO induced a significant G0/G1 phase accumulation (**Figures 2A, C**). Similarly, MK-8353 treatment arrested WT cells in the G0/G1 phase. However, this effect was stronger in SC3KO cells (G0/G1: 81.4% vs. 71.9%), suggesting that loss of SCAMP3 enhances sensitivity to ERK1/2 inhibition at the G1 restriction point (**Figures 2A, C**). As shown in **Figures 2E**, MK-8353 increased cyclin D1 and reduced cyclin E1 and B1 in WT cells, a pattern compatible with G1 accumulation and reduced progression to S phase. In particular, SC3KO cells displayed upregulation of cyclin B1 under both vehicle and MK-8353 conditions, suggesting that SCAMP3 loss disrupts cyclin regulation (**Figure 2E**).

**Figure 2:**
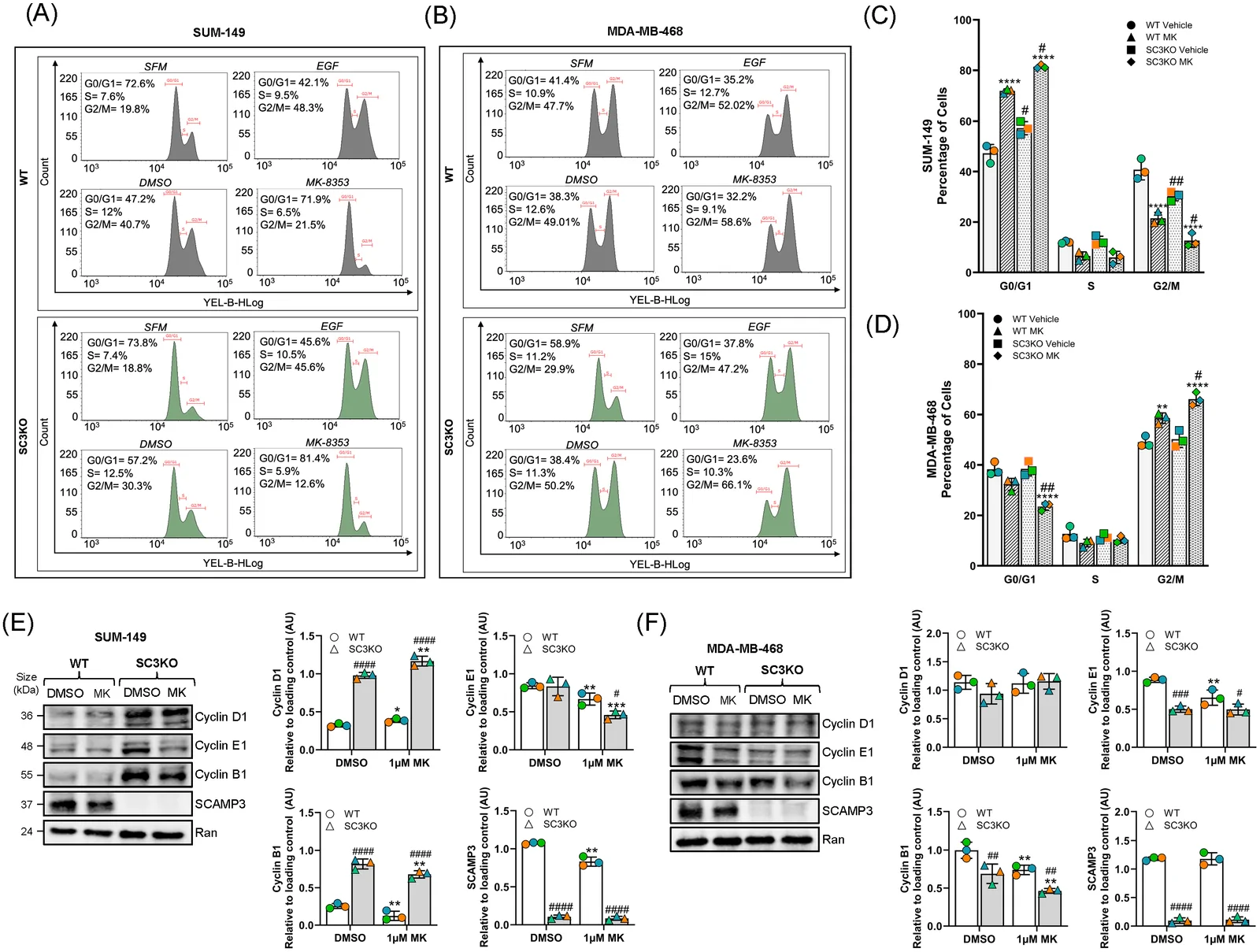
SCAMP3 loss regulates ERK1/2-dependent cell cycle control in TNBC cells. Cell cycle profiles of **(A)** SUM-149 and **(B)** MDA-MB-468 wild-type (WT) and SCAMP3 knockout (SC3KO) cells after 24 h of treatment with serum-free medium (SFM), EGF (10 ng/mL), DMSO (vehicle), or 1 μM MK-8353, assessed by flow cytometry. SFM and EGF were included as functional controls to verify cell cycle arrest and mitogenic responsiveness, respectively. Percentages of cells in G0/G1, S, and G2/M phases are indicated within each histogram. Quantification of cell cycle distribution in **(C)** SUM-149 and **(D)** MDA-MB-468 WT and SC3KO cells. **(E, F)** Immunoblots and densitometric quantification of cyclins in SUM-149 and MDA-MB-468 WT and SC3KO cells treated with DMSO or MK-8353 (1 µM) for 24 h. Ran was used as a loading control. Data were analyzed using two-way ANOVA with Tukey´s multiple comparison test. Individual data points represent independent biological replicates and are reported as mean ± SD (n = 3). *P ≤ 0.05, **P < 0.01, ***P < 0.001 vs. DMSO within the same genotype; ^#^P < 0.05, ^##^P < 0.01, ^###^P < 0.001, ^####^P < 0.0001 for WT vs. SC3KO under the indicated condition.

MDA-MB-468 SC3KO cells did not show significant distribution changes but did show reduced cyclins E1 and B1 (**Figures 2B, D, F**). Following MK-8353 treatment, both WT and SC3KO MDA-MB-468 cells showed G2/M phase accumulation with a significantly higher fraction in SC3KO cells (66.1% vs. 58.6%). MK-8353 also reduced the expression of cyclins E1 and B1 in both genotypes. Taken together, these results indicate that the SCAMP3 loss is associated with impaired ERK1/2-dependent G1-S transition in SUM-149 cells, whereas the cell cycle machinery in MDA-MB-468 cells partially compensates for its loss.

### 3.3 SCAMP3 loss affects the apoptotic response to MK-8353

We then examined whether SCAMP3 loss alters apoptotic responses to ERK1/2 inhibition. To this end, SUM-149 and MDA-MB-468 cells were treated with vehicle or MK-8353 for 24h, stained with annexin V and propidium iodide, and analyzed by flow cytometry. Flow cytometry analysis showed that SCAMP3 knockout alone increased the percentage of early apoptotic cells compared to WT SUM-149 cells (**Figures 3A, C**). At the molecular level, SC3KO cells showed reduced total caspase-3, and MK-8353 further induced robust cleavage of PARP-1 (**Figure 3E**). To assess whether necroptosis contributes to cell death after SCAMP3 knockout or ERK1/2 inhibition, we monitored the levels of total and p-MLKL, the terminal effector of necroptosis (40). Both total MLKL and p-MLKL were downregulated in SC3KO cells, resulting in an unaltered p-MLKL/total MLKL ratio. Surprisingly, MK-8353 does not alter the percentage of early apoptosis in WT cells or the expression of cell death-associated proteins.

**Figure 3:**
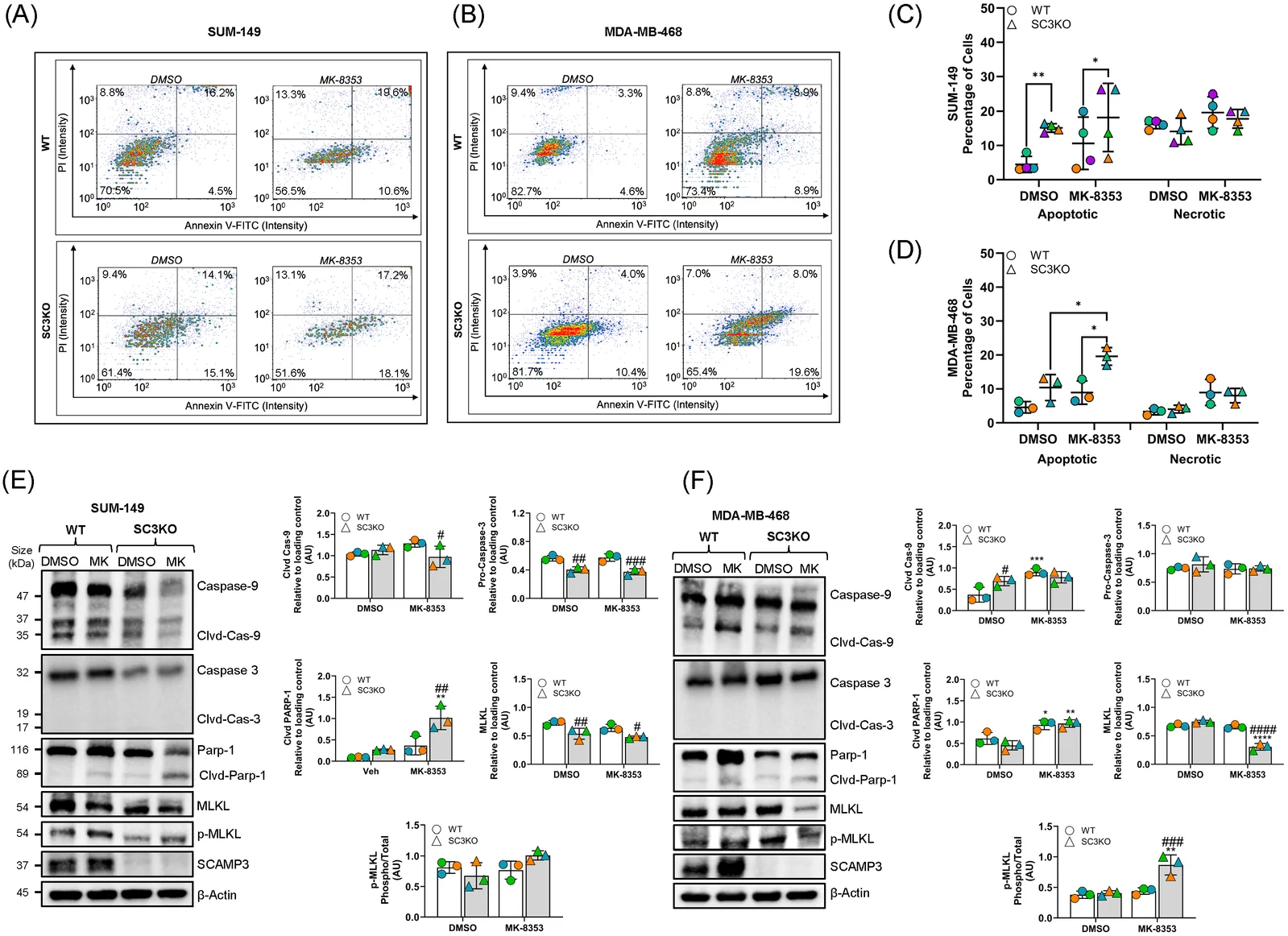
SCAMP3 loss modulates apoptosis and cell death pathways induced by MK-8353. Representative Annexin V-FITC/PI flow cytometry plots of **(A)** SUM-149 and **(B)** MDA-MB-468 wild-type (WT) and SCAMP3 knockout (SC3KO) cells after 24 h treatment with DMSO (vehicle) or MK-8353 (2 μM). **(C, D)** Quantification of apoptotic and Annexin V^+^/PI^+^ populations in SUM-149 and MDA-MB-468 cells. Annexin V^+^/PI^+^ (late apoptotic/necrotic cells) are labeled as “Necrotic”. **(E, F)** Immunoblots and densitometric quantification of cell death proteins in SUM-149 and MDA-MB-468 WT and SC3KO cells treated with DMSO or MK-8353 (2 μM) for 24 h. β-actin was used as a loading control. The phospho/total ratio was calculated as (phosphoprotein normalized to loading control)/(total protein normalized to loading control). Data were analyzed using two-way ANOVA with Tukey´s multiple comparison test. Individual data points represent independent biological replicates and are reported as mean ± SD (n = 3 or 4). *P ≤ 0.05, **P < 0.01, ***P < 0.001, ****P < 0.0001 vs. DMSO within the same genotype; (#) indicates significance for WT vs. SC3KO under the indicated condition.

Contrary to our observations in SUM-149 cells, the flow cytometry analysis did not show any significant differences between SC3KO and parental MDA-MB-468 cells (**Figures 3B, D**). However, an increase in cleaved caspase-9 was observed (**Figure 3F**). The same results were observed in WT cells treated with MK-8353. In contrast, MK-8353 significantly increased the percentage of early apoptotic SC3KO cells and promoted PARP-1 cleavage. MK-8353 also reduced total MLKL but increased its phosphorylation. However, this molecular response was not translated into an increase in the percentage of necrotic cells.

### 3.4 SCAMP3 loss impairs MAPK pathway signaling and enhances the effects of ERK1/2 inhibition

We evaluated whether SCAMP3 loss modifies the early signaling response to ERK1/2 inhibition. Immunoblot analysis of MAPK pathway proteins in SUM-149 cells showed that the knockout of SCAMP3 reduced basal expression of total B-Raf and decreased the phosphorylation of MEK1/2 and ERK1/2, as well as of downstream transcription factors ELK-1 and c-Fos (**Figures 4A, B**; **Supplementary Figure 1A**), indicating impaired signal propagation across the MAPK cascade. After EGF stimulation, WT cells showed an expected increase in p-MEK1/2, p-ERK1/2, and p-ELK1, consistent with pathway activation. This response was attenuated in SC3KO cells; however, p-MEK1/2 levels remained unchanged. Although MK-8353 inhibited p-ERK1/2 and downstream effectors in WT cells, the increase in p-MEK1/2 observed after pathway activation remained. The knockout of SCAMP3 further reduced p-ERK1/2 and abrogated the EGF-induced response in p-MEK1/2. Immunoblots of total protein showed that the expression of B-Raf and ELK-1 is downregulated in SC3KO (**Supplementary Figure 1A**). A similar reduction in total B-Raf and p-ERK1/2 was observed in SC3KO MDA-MB-468 cells (**Figures 4C, D**; **Supplementary Figure 1B**). MK-8353 treatment increased p-MEK1/2 in both WT and SC3KO cells. Although the knockout of SCAMP3 reduced residual p-ERK1/2 after treatment, the phosphorylation of transcription factors was not affected. Interestingly, p-c-Fos was not affected across conditions, suggesting its activation is independent of the ERK1/2 pathway in these cells.

**Figure 4:**
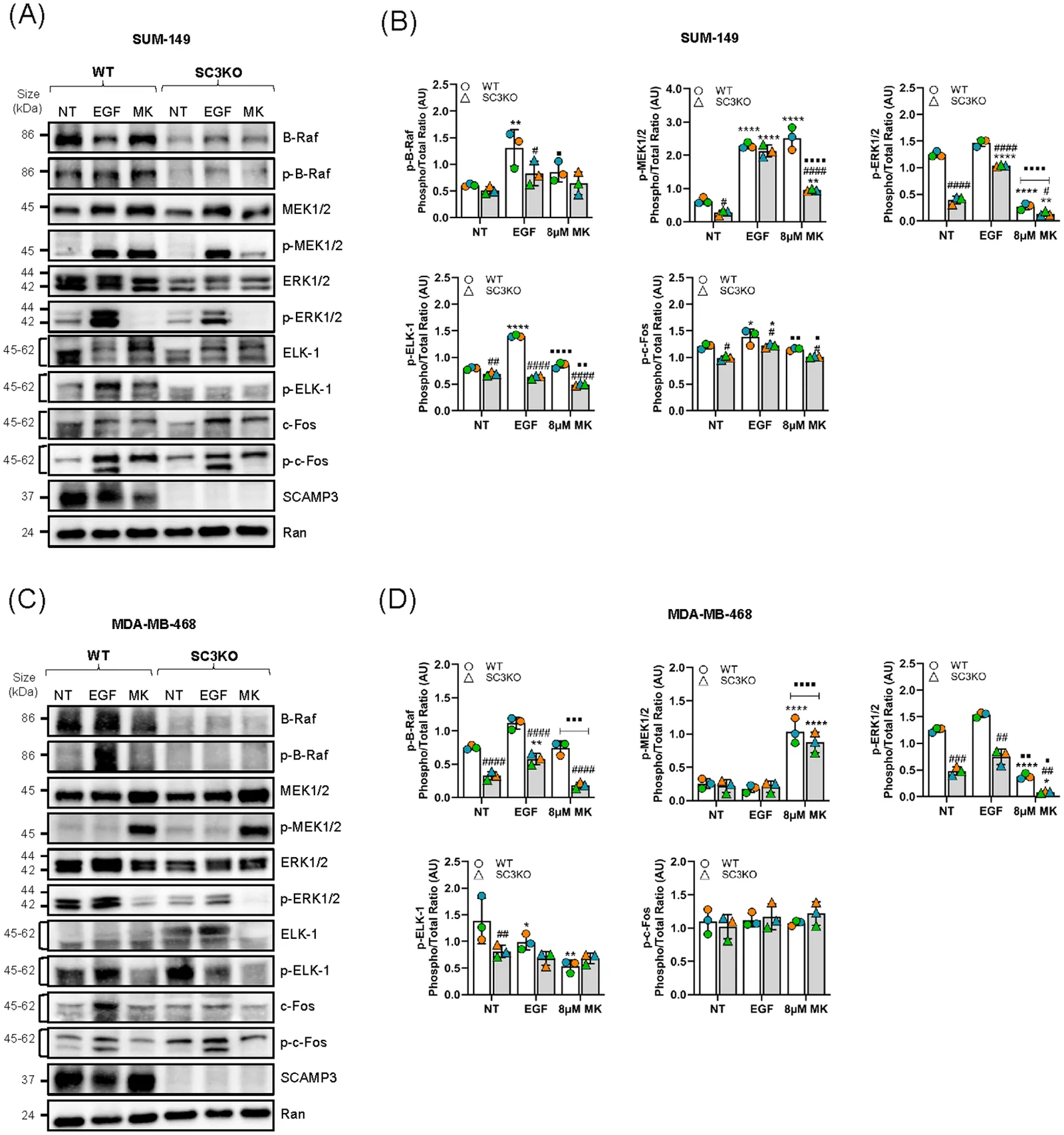
SCAMP3 loss modulates RAF-MEK-ERK signaling in response to ERK1/2 inhibition. SUM-149 and MDA-MB-468 wild-type (WT) and SCAMP3 knockout (SC3KO) cells were analyzed under basal conditions (NT), after EGF stimulation (10 ng/mL, 30 min), or following treatment with MK-8353 (8 μM, 2 h). **(A)** Immunoblots of MAPK pathway proteins in SUM-149 WT and SC3KO cells. **(B)** Densitometric quantification of phosphorylated/total protein ratios in SUM-149 cells. **(C)** Immunoblots of MAPK pathway proteins in MDA-MB-468 WT and SC3KO cells. **(D)** Densitometric quantification of phosphorylated/total protein ratios in MDA-MB-468 cells. Ran was used as a loading control. Data were analyzed using a two-way ANOVA with Tukey´s multiple-comparison test. Individual data points represent independent biological replicates and are reported as mean ± SD (n = 3). *P ≤ 0.05, **P<0.01, ****P<0.0001 vs. NT within the same genotype; ^#^P < 0.05, ^##^P < 0.01, ^###^P < 0.001, and ^####^P < 0.0001 for WT vs. SC3KO under the indicated condition; (▪) indicates significance for EGF vs. MK-8353.

### 3.5 SCAMP3 supports the nuclear accumulation of active ERK1/2

To further define how SCAMP3 regulates nuclear ERK1/2 signaling, we analyzed the subcellular distribution of SCAMP3 and active ERK1/2 (p-ERK1/2) in SUM-149 cells using immunofluorescence (**Figure 5A**). **Figure 5B** presents the quantification of total cellular fluorescence signal of p-ERK1/2 or SCAMP3 normalized to untreated WT cells. After EGF stimulation, WT cells exhibited a significant increase in total cellular SCAMP3 and p-ERK1/2, accompanied by increased SCAMP3/p-ERK1/2 colocalization within perinuclear and nuclear regions (**Figures 5A–C**). MK-8353 reduced SCAMP3 and p-ERK1/2 in WT cells and abolished detectable nuclear colocalization of SCAMP3 and p-ERK1/2. Interestingly, the SCAMP3 signal increased upon pathway activation.

**Figure 5:**
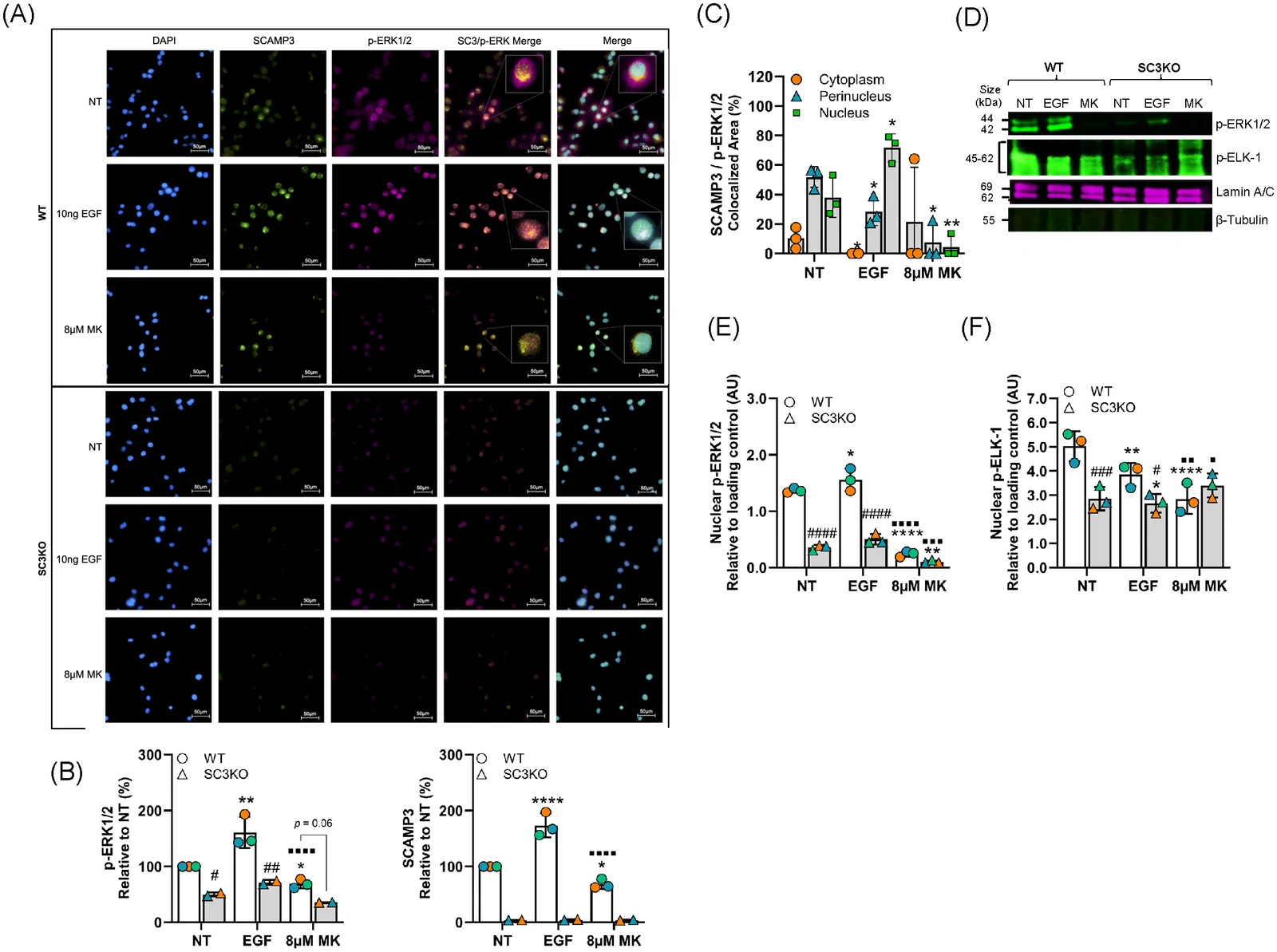
SCAMP3 supports nuclear accumulation of active ERK1/2 in SUM-149 cells. **(A)** Immunofluorescence analysis of SCAMP3 (green), p-ERK1/2 (magenta), and nuclei (DAPI, blue) in wild-type (WT) and SCAMP3 knockout (SC3KO) SUM-149 cells under basal conditions (NT), after EGF stimulation (10 ng/mL, 30 min), or following MK-8353 treatment (8 μM, 2 h). Insets highlight SCAMP3/p-ERK1/2 colocalization (yellow/orange) in merged images. **(B)** Quantification of p-ERK1/2 and SCAMP3 fluorescence intensity expressed as a percentage of total cellular signal normalized to WT NT cells. **(C)** Quantification of SCAMP3/p-ERK1/2 colocalization within cytoplasmic, perinuclear, and nuclear compartments in WT cells. **(D)** Immunoblots of nuclear fractions. **(E, F)** Densitometric quantification of p-ERK1/2 (T202; Y204), p-ELK-1 (S358), and SCAMP3 in WT and SC3KO cells. Lamin A/C and β-tubulin were used as nuclear and cytosolic controls, respectively. The data were analyzed using two-way ANOVA with Tukey´s multiple comparison test. Individual data points represent independent biological replicates and are reported as mean ± SD (n = 3). *P ≤ 0.05, **P < 0.01, ***P < 0.001, ****P < 0.0001 vs. NT within the same genotype; (#) indicates significance for WT vs. SC3KO under the indicated condition; (▪) indicates significance for EGF vs. MK-8353.

Nuclear fractionation further supported the immunofluorescence findings. As a quality control for fractionation, β-tubulin was not detected in the nuclear-enriched fractions. In WT cells, EGF increased nuclear p-ERK1/2, while MK-8353 reduced both p-ERK1/2 and p-ELK-1 (**Figures 5D–F**). In SC3KO cells, nuclear p-ERK1/2 and p-ELK-1 levels were reduced, and MK-8353 further suppressed residual nuclear p-ERK1/2. Because importin-7 participates in the nuclear translocation of active ERK1/2 (41), we assessed its expression in SUM-149 cells. SC3KO cells showed reduced importin-7 protein levels under basal, EGF-stimulated, and MK-8353-treated conditions (**Supplementary Figure 2**). Collectively, these findings suggest that SCAMP3 loss is associated with reduced nuclear accumulation and signaling activity of ERK1/2, and a modest reduction in importin-7 expression.

### 3.6 SCAMP3 loss suppresses tumor growth and enhances the response to ERK1/2 inhibition *in vivo*

Finally, we examined the role of SCAMP3 in tumor growth and response to ERK1/2 inhibition *in vivo* using orthotopic SUM‑149 WT and SC3KO xenografts. Mice were treated with vehicle or MK‑8353 (30 mg/kg BID) (**Figure 6A**). Body weight remained stable across all groups, indicating that MK‑8353 was well tolerated (**Figure 6B**). SC3KO tumors grew significantly more slowly than WT tumors, and MK-8353 further delayed tumor progression in both genotypes, with the greatest inhibition observed in SC3KO tumors (**Figure 6C**). The endpoint tumor weights were consistent with the differences observed in tumor volume, with the greatest reduction observed in SC3KO tumors treated with MK 8353 (**Figure 6D**).

**Figure 6:**
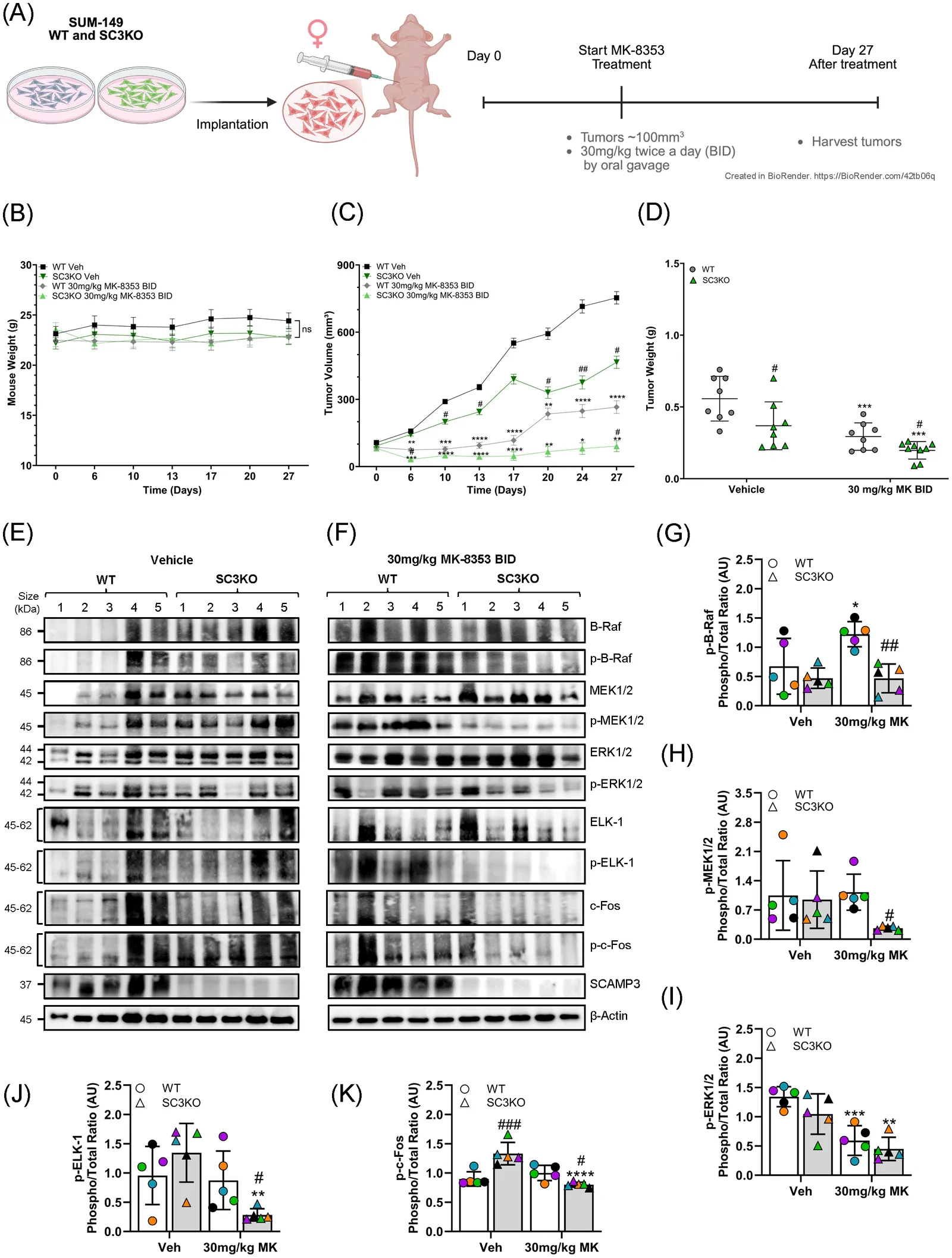
SCAMP3 loss enhances the antitumor efficacy of ERK1/2 inhibition in SUM-149 xenografts. **(A)** Experimental design of the *in vivo* study. SUM-149 wild-type (WT) and SCAMP3 knockout (SC3KO) cells were orthotopically implanted into SCID mice. Animals were treated with either vehicle or MK-8353 (30 mg/kg, BID, by oral gavage) for 27 days, n=10/group. **(B)** Mouse body weight during treatment. **(C)** Tumor growth curves of WT and SC3KO xenografts treated with vehicle or MK-8353. **(D)** Endpoint tumor weights. **(E)** Immunoblots of MAPK pathway proteins in tumor lysates from vehicle. **(E, F)** Immunoblots of MAPK pathway proteins in tumor lysates from vehicle and MK-8353-treated mice. **(G–K)** Densitometric quantification of phospho/total protein ratios. β-actin was used as a loading control. Individual data points represent independent biological replicates. Data are reported as mean ± SD (n = 5/group). Tumor growth curves and mouse body weight measurements data are presented as mean ± SEM. *P ≤ 0.05, **P < 0.01, ***P < 0.001, ****P < 0.0001 vs. vehicle within each genotype; (#) indicates significance for WT vs. SC3KO under the indicated condition.

Immunoblot analyses of tumor lysates under vehicle treatment showed that SC3KO tumors maintained phosphorylation levels of p-B-RAF, p-MEK1/2, p-ERK1/2, and p-ELK-1 comparable to those observed in WT tumors, while displaying increased p‑c‑Fos (**Figures 6E–J**). SC3KO tumors also showed increased total B-Raf and reduced ELK-1 levels (**Supplementary Figure 3**). In WT tumors, MK-8353 reduced p-ERK1/2 without affecting downstream proteins, whereas p-B-Raf increased, suggesting incomplete pathway inhibition and activation of a potential feedback mechanism. In contrast, treatment with MK-8353 in SC3KO tumors led to greater suppression of the pathway. Unlike the *in vitro* findings, SCAMP3 protein levels were not affected by MK-8353, suggesting that its abundance may be less responsive to sustained ERK1/2 inhibition.

We also evaluated MDA-MB-468 WT and SC3KO xenografts. In this model, SCAMP3 silencing did not significantly affect mouse weight, tumor volume, or endpoint tumor weight (**Supplementary Figures S4A–C**). Consistent with *in vitro* protein expression, SC3KO tumors reduced p-ERK1/2 (**Supplementary Figures S4D–E**). Overall, these data suggest that SCAMP3 loss not only reduces tumor growth but also enhances the inhibitory effect of MK-8353 in MAPK wild-type TNBC tumors.

## 4 Discussion

This study identifies SCAMP3 as a regulator of RAF-MEK-ERK signaling that modulates the biological response of TNBC cells to ERK1/2 inhibition. Our findings demonstrate that SCAMP3 sustains ERK1/2 signaling output, supports the nuclear accumulation of active ERK1/2, and promotes ERK1/2-dependent cell-cycle progression, and survival. These observations extend our previous work by showing that SCAMP3 influences not only EGFR trafficking but also the downstream cellular response to pharmacologic ERK1/2 inhibition.

SCAMP3 localizes to endosomal membranes and regulates EGFR trafficking by coordinating receptor recycling and degradation, sustaining downstream signaling, and supporting cancer cell proliferation and migration (25, 26). Our previous work showed that in TNBC, SCAMP3 positively regulates EGFR signaling and its loss reduces the gene expression of RAS/MAPK components. Adaptors and signaling mediators associated with EGFR, including *SHC1* and *EPS8*, were also reduced following SCAMP3 depletion. This finding is consistent with reports identifying EPS8 as a key EGFR effector in prostate cancer (25, 26, 42). Phosphoproteomic analysis further showed that SCAMP3 depletion disrupts ERK1/2 activation dynamics and dysregulates the phosphorylation of MAPK regulators and ERK1/2 substrates (29). These observations are in agreement with the general view that trafficking adaptors actively regulate oncogenic signaling rather than simply control receptor turnover (43–45). For example, adaptor proteins such as GRB2 and SHC maintain endosomal RAS-ERK1/2 signaling, while ubiquitin ligases such as CBL promote receptor downregulation and limit the duration of the signal (46–48). A similar role has been described for the endocytic scaffold HIP1, which enhances receptor-driven ERK1/2 activation in prostate cancer (45). A central insight from this study is that SCAMP3 does not determine acute cytotoxic sensitivity to ERK1/2 inhibition but instead modulates the biological response to pathway suppression. In the TNBC models examined here, SCAMP3 perturbation produced only modest changes in IC_50_ values. Our previous observation that SCAMP3 knockdown decreases ERK1/2 phosphorylation in these cells also helps explain the modest changes observed in cell viability assays (26, 29). Therefore, IC_50_ values alone underestimate SCAMP3’s contribution to the ERK1/2 inhibitor response in these models.

Mechanistically, SCAMP3 appears to sustain efficient propagation of the MAPK signaling in RAS/RAF wild-type SUM-149 cells, where ERK1/2 activation depends heavily on receptor tyrosine kinase input (47). SCAMP3 loss reduced phosphorylation of MEK1/2, ERK1/2, and downstream transcription factors, indicating weaker signaling transmission through the MEK-ERK cascade. Furthermore, SC3KO reduced total B-Raf and ELK-1, consistent with our earlier finding that SCAMP3 loss downregulates *BRAF*, *MAP2K1* (MEK1), and *ELK1* transcripts, thereby weakening the molecular framework that sustains MAPK signaling (26). The attenuated response to EGF in SC3KO cells further supports our and others’ reports that SCAMP3 contributes to EGFR-driven activation of the MAPK-ERK1/2 pathway (26, 29, 34). MDA-MB-468 cells, which harbor strong intrinsic MAPK activation, showed a distinct signaling organization that helps explain why SCAMP3 loss had a weaker overall effect in this model.

The increased of p-MEK1/2 after MK-8353 is consistent with activation of upstream compensatory feedback pathways, in which relief of ERK1/2-dependent negative feedback allows MEK phosphorylation despite reduced downstream ERK1/2 output (10). In the same model, ERK1/2 inhibition did not affect p-c-Fos in either genotype, indicating that downstream transcription factor phosphorylation was not tightly coupled to ERK1/2 inhibition under these conditions. Because c-Fos can also receive input from parallel MAPK pathways, including JNK and p38, these findings suggest that SCAMP3 does not control the compensatory increase in p-MEK1/2 itself (49). Instead, SCAMP3 appears to support efficient transmission of MAPK signaling from upstream components to ERK1/2 and downstream nuclear effectors. A similar principle has been described for scaffold proteins such as CNKSR1, which can influence MEK inhibitor response in KRAS-driven models by coordinating MAPK with other survival networks to promote treatment resistance (50). Although our data do not establish an equivalent mechanism for SCAMP3, they support the idea that SCAMP3 modifies how tumor cells respond to ERK1/2 pathway inhibition. Interestingly, MK-8353 also reduced SCAMP3 protein levels in some experiments, although this effect was not consistently observed across all experimental conditions. Previous studies demonstrated that EGFR-MAPK signaling positively regulates SCAMP3 expression, while SCAMP3 deficiency impairs mTORC1 signaling and disrupts autophagic flux, supporting a potential feedback relationship between ERK1/2-mTORC1 signaling, SCAMP3 expression, and endolysosomal pathways (26, 29). Although the mechanism underlying the reduction in SCAMP3 following ERK1/2 inhibition remains unknown, altered protein turnover through lysosomal and/or proteasomal degradation represents one possible explanation. However, additional studies will be required to determine whether these changes reflect altered protein stability, degradation, or transcriptional regulation.

These signaling changes prompted us to examine whether SCAMP3 influences the nuclear organization of ERK1/2 signaling. Nuclear translocation of active ERK1/2 is required for phosphorylation and activation of downstream transcription factors such as ELK-1 and AP-1 (41). In parental SUM-149 cells, SCAMP3 and p-ERK1/2 showed prominent colocalization in perinuclear regions after receptor stimulation, whereas SCAMP3 loss was associated with reduced nuclear p-ERK1/2 and p‑ELK, and lower importin-7 expression. We previously reported that SCAMP3 knockout reduces phosphorylation of the nucleoporins NUP153 and NUP214, which have been associated with ERK1/2 nuclear passage (29, 51). Together, these findings support that SCAMP3 maintains the nuclear accumulation and signaling output of active ERK. However, they do not demonstrate a direct interaction between SCAMP3 and the nuclear import machinery, and the precise mechanism remains to be elucidated.

The reduction in nuclear ERK1/2 signaling may partially explain the cell-cycle defects observed after SCAMP3 loss. In SUM-149 cells, SC3KO increased the G_0_/G1 fraction and strengthened the arrest in G1 after ERK1/2 inhibition despite elevated cyclin D1. This result suggests that cyclin D1 accumulation alone was not sufficient to sustain productive progression into S phase. Nuclear ERK1/2 does not simply induce cyclin D1 expression; it also links cyclin D1 to cyclin-dependent kinase activation and S-phase entry (8, 52). When nuclear ERK1/2 signaling declines, cyclin D1 can accumulate without driving G1/S progression, thus favoring checkpoint engagement. In MDA-MB-468 cells, by contrast, ERK1/2 inhibition increased the accumulation of cells at G_2_/M, particularly in SC3KO cells, which is consistent with the requirement for sustained ERK1/2 signaling during late cell cycle progression in RAS-driven cancers (53, 54). Other groups also reported that SCAMP3 loss leads to the accumulation of melanoma cells in the S and G_2_/M phases and to G1 arrest in hepatocellular carcinoma cells. Together, these findings indicate that SCAMP3 modulates ERK1/2-dependent checkpoint control, but the cell-cycle phase most affected depends on the signaling state of the models.

SCAMP3 loss also altered cell‑fate responses to ERK1/2 inhibition, sensitizing TNBC cells to apoptosis. Although cleaved caspase-3 was not detected, the reduction in procaspase 3 observed in SUM-149 SC3KO cells may reflect the downregulation of *CASP3* gene expression reported previously (26). On the other hand, our data provide limited evidence for the activation of canonical necroptosis. Although changes in total and phosphorylated MLKL were observed, they were not accompanied by a necrotic phenotype, indicating that MLKL regulation alone is insufficient to infer necroptotic cell death. Beyond its established role in necroptosis, MLKL has also been implicated in endosomal trafficking and autophagy through mTOR pathway. Therefore, the observed changes in MLKL may instead reflect broader changes in cellular stress responses rather than the activation of a canonical necroptotic program. Consistent with our previous findings that SCAMP3 deficiency impairs mTOR signaling and disrupts autophagy flux under ERK1/2 inhibition, it is reasonable to hypothesize that loss of SCAMP3 may shift the cellular response away from adaptive survival mechanisms toward apoptosis (29, 55). However, additional studies will be required to determine the precise molecular mechanisms underlying apoptosis, the contribution of autophagy, and other MLKL-associated pathways to the response of SCAMP3-deficient cells to ERK1/2 inhibition.

Our xenograft studies support the *in vivo* relevance of SCAMP3-dependent signaling. These findings align with the reduced ERK1/2 signaling and impaired proliferation observed *in vitro*, suggesting that SCAMP3 contributes to tumor maintenance. The inhibitory effect of SCAMP3 loss on tumor growth was more pronounced than that observed in our previous study using the same SUM-149 xenograft model (26). We believe this difference likely reflects improvements in the experimental design rather than differences in the biological phenotype. In the earlier study, several animals were lost before the planned experimental endpoint for reasons unrelated to the experimental conditions, reducing sample size and increasing variability in tumor measurements. In contrast, the present study included a larger cohort, allowing clearer separation of the WT and SC3KO growth curves. Importantly, despite these quantitative differences, both studies consistently demonstrate that SCAMP3 loss delays SUM-149 tumor growth, supporting a role for SCAMP3 in TNBC progression.

However, the tumor signaling profile did not fully replicate our *in vitro* observations. In cultured SUM-149 cells, SCAMP3 loss reduced the activation of multiple RAF–MEK–ERK pathway components, whereas vehicle-treated SC3KO xenografts maintained baseline phosphorylation levels of B-RAF, MEK1/2, ERK1/2, and ELK-1 comparable to WT tumors. Furthermore, while MK-8353 induced a dose-dependent decrease in SCAMP3 protein levels *in vitro*, SCAMP3 expression remained unchanged *in vivo*. Several factors may contribute to these differences. The tumor microenvironment provides stromal-derived growth factors, cytokines, and extracellular matrix interactions that are absent in standard culture conditions, which can compensate for signal defects. Variations in drug exposure, pharmacokinetics, protein turnover, and compensatory survival loops *in vivo* may also contribute (26, 56, 57). Additionally, endpoint analyses of whole-tumor lysates fail to fully capture spatial alterations in ERK signaling. Because our *in vitro* data demonstrate that SCAMP3 loss preferentially affects the nuclear accumulation of active ERK1/2, localized nuclear signaling defects may persist *in vivo* even when total pathway phosphorylation appears unchanged. For instance, the elevated p-c-Fos levels observed in SC3KO tumors may reflect the activation of alternative stress-activated kinases capable of sustaining Activator protein-1 (AP-1) activity independently of canonical ERK1/2 (58). Notably, while MK-8353 failed to alter downstream p-ELK-1 or p-c-Fos levels in WT tumors, it significantly reduced the phosphorylation of both transcription factors in SC3KO tumors. This suggests that the discrepancies between our *in vitro* and *in vivo* signaling profiles likely reflect the complexity of the tumor architecture, but the heightened drug sensitivity confirms that SCAMP3 loss compromises pathway efficiency.

The MDA-MB-468 xenografts further support the overall framework. Although SCAMP3 loss reduced p-ERK1/2, tumor growth was not significantly affected. The absence of ERK1/2 inhibitor treatment and the small cohort size limit the interpretation of this model. Nevertheless, these findings are consistent with our *in vitro* observations, where SCAMP3 depletion reduced ERK1/2 signaling but produced less pronounced biological effects in the N-RAS-overexpressing model than in SUM-149 cells. Thus, stronger intrinsic MAPK activation may partially buffer the consequences of SCAMP3 loss.

Although the TNBC models used in this study represent distinct MAPK signaling contexts, they do not encompass the full molecular heterogeneity of the disease. In addition, while our findings demonstrate that SCAMP3 supports the nuclear accumulation of active ERK1/2, the precise molecular mechanisms underlying this process remain to be defined. Future studies incorporating rescue experiments, additional TNBC models, and mechanistic analyses of ERK nuclear import will further refine our understanding of SCAMP3 function. Finally, because the xenograft studies were conducted in immunodeficient mice, the potential contribution of the tumor immune microenvironment to SCAMP3-dependent signaling could not be evaluated in the present study.

Despite these limitations, the present findings consistently demonstrate SCAMP3 is an important regulator of ERK1/2 signaling and therapeutic response in TNBC. In summary, our findings extend previous evidence linking SCAMP3 to ERK1/2-driven programs in TNBC by showing that SCAMP3 supports RAF-MEK-ERK1/2 signaling, maintains nuclear ERK1/2 activity, and influences ERK1/2-dependent cell cycle and apoptotic responses. Although SCAMP3 perturbation produced only modest changes in acute *in vitro* sensitivity to MK-8353, SCAMP3 loss enhanced the suppressive effects of ERK1/2 inhibition on MAPK signaling and tumor growth in RAS/RAF wild-type TNBC xenografts. These insights position the SCAMP3-ERK1/2 axis as a relevant modulator of TNBC behavior and support further investigation of SCAMP3 in strategies to improve ERK1/2-targeted therapy, particularly in tumors where receptor-proximal signaling remains a dominant driver of ERK activity.

## Data Availability

The original contributions presented in the study are included in the article/**Supplementary Material**. Further inquiries can be directed to the corresponding author.

## References

[1] Dogra AK Prakash A Gupta S Gupta M . Prognostic significance and molecular classification of triple negative breast cancer: a systematic review. Eur J Breast Health. (2025) 21:101–14. doi: 10.4274/ejbh.galenos.2025.2024-10-2 40028895 PMC11934825

[2] Xiong N Wu H Yu Z . Advancements and challenges in triple-negative breast cancer: a comprehensive review of therapeutic and diagnostic strategies. Front Oncol. (2024) 14:1405491. doi: 10.3389/fonc.2024.1405491 38863622 PMC11165151

[3] O'Reilly D Sendi MA Kelly CM . Overview of recent advances in metastatic triple negative breast cancer. World J Clin Oncol. (2021) 12:164–82. doi: 10.5306/wjco.v12.i3.164 33767972 PMC7968109

[4] Bianchini G De Angelis C Licata L Gianni L . Treatment landscape of triple-negative breast cancer - expanded options, evolving needs. Nat Rev Clin Oncol. (2022) 19:91–113. doi: 10.1038/s41571-021-00565-2 34754128

[5] Cortes J Rugo HS Cescon DW Im SA Yusof MM Gallardo C . Pembrolizumab plus chemotherapy in advanced triple-negative breast cancer. N Engl J Med. (2022) 387:217–26. doi: 10.1056/NEJMoa2202809 35857659

[6] Fei J Guo Y . MAPK/ERK signaling in tumorigenesis: mechanisms of growth, invasion, and angiogenesis. Excli J. (2025) 24:854–79. doi: 10.17179/excli2025-8479 40964172 PMC12436680

[7] Durrant DE Morrison DK . Targeting the Raf kinases in human cancer: the Raf dimer dilemma. Br J Cancer. (2018) 118:3–8. doi: 10.1038/bjc.2017.399 29235562 PMC5765234

[8] Yoshizawa R Sako Y . Cell cycle dependence of ERK activation dynamics is regulated by PI3K and PAK1 signaling. Sci Rep. (2025) 15:27688. doi: 10.1038/s41598-025-13686-w 40731057 PMC12307696

[9] Guo YJ Pan WW Liu SB Shen ZF Xu Y Hu LL . ERK/MAPK signalling pathway and tumorigenesis. Exp Ther Med. (2020) 19:1997–2007. doi: 10.3892/etm.2020.8454 32104259 PMC7027163

[10] Ram A Murphy D DeCuzzi N Patankar M Hu J Pargett M . A guide to ERK dynamics, part 1: mechanisms and models. Biochem J. (2023) 480:1887–907. doi: 10.1042/bcj20230276 38038974 PMC10754288

[11] Bahar ME Kim HJ Kim DR . Targeting the RAS/RAF/MAPK pathway for cancer therapy: from mechanism to clinical studies. Signal Transduction Targeted Ther. (2023) 8:455. doi: 10.1038/s41392-023-01705-z 38105263 PMC10725898

[12] Ma J Dong C Cao YZ Ma BL . Dual target of EGFR and mTOR suppresses triple-negative breast cancer cell growth by regulating the phosphorylation of mTOR downstream proteins. Breast Cancer (Dove Med Press). (2023) 15:11–24. doi: 10.2147/bctt.S390017 36691572 PMC9864233

[13] Banushi B Joseph SR Lum B Lee JJ Simpson F . Endocytosis in cancer and cancer therapy. Nat Rev Cancer. (2023) 23:450–73. doi: 10.1038/s41568-023-00574-6 37217781

[14] Jiang W Wang X Zhang C Xue L Yang L . Expression and clinical significance of MAPK and EGFR in triple-negative breast cancer. Oncol Lett. (2020) 19:1842–8. doi: 10.3892/ol.2020.11274 32194678 PMC7038935

[15] Kwon J Helfman DM Kim MY . AKT drives sustained motility following MEK inhibition via promoting SNAIL and AXL in MDA-MB-231 LM2. Biochem Biophys Res Commun. (2020) 528:92–8. doi: 10.1016/j.bbrc.2020.05.043 32456793

[16] Ethier SP Guest ST Garrett-Mayer E Armeson K Wilson RC Duchinski K . Development and implementation of the SUM breast cancer cell line functional genomics knowledge base. NPJ Breast Cancer. (2020) 6:30. doi: 10.1038/s41523-020-0173-z 32715085 PMC7374090

[17] Kim RK Suh Y Yoo KC Cui YH Kim H Kim MJ . Activation of KRAS promotes the mesenchymal features of basal-type breast cancer. Exp Mol Med. (2015) 47:e137. doi: 10.1038/emm.2014.99 25633745 PMC4314588

[18] Shi Y Jin J Ji W Guan X . Therapeutic landscape in mutational triple negative breast cancer. Mol Cancer. (2018) 17:99. doi: 10.1186/s12943-018-0850-9 30007403 PMC6046102

[19] Zheng ZY Tian L Bu W Fan C Gao X Wang H . Wild-type N-Ras, overexpressed in basal-like breast cancer, promotes tumor formation by inducing IL-8 secretion via JAK2 activation. Cell Rep. (2015) 12:511–24. doi: 10.1016/j.celrep.2015.06.044 26166574 PMC4512851

[20] Kumar A Ahmad A Vyawahare A Khan R . Membrane trafficking and subcellular drug targeting pathways. Front Pharmacol. (2020) 11:629. doi: 10.3389/fphar.2020.00629 32536862 PMC7267071

[21] Peterson AF Ingram K Huang EJ Parksong J McKenney C Bever GS . Systematic analysis of the MAPK signaling network reveals MAP3K-driven control of cell fate. Cell Syst. (2022) 13:885–94. doi: 10.1016/j.cels.2022.10.003 36356576 PMC9923805

[22] Falguières T Castle D Gruenberg J . Regulation of the MVB pathway by SCAMP3. Traffic. (2012) 13:131–42. doi: 10.1111/j.1600-0854.2011.01291.x 21951651

[23] Meister M Tomasovic A Banning A Tikkanen R . Mitogen-activated protein (MAP) kinase scaffolding proteins: a recount. Int J Mol Sci. (2013) 14:4854–84. doi: 10.3390/ijms14034854 23455463 PMC3634400

[24] Yuan L Barbash S Kongsamut S Eishingdrelo A Sakmar TP Eishingdrelo H . 14-3-3 signal adaptor and scaffold proteins mediate GPCR trafficking. Sci Rep. (2019) 9:11156. doi: 10.1038/s41598-019-47478-w 31371790 PMC6673703

[25] Aoh QL Castle AM Hubbard CH Katsumata O Castle JD . SCAMP3 negatively regulates epidermal growth factor receptor degradation and promotes receptor recycling. Mol Biol Cell. (2009) 20:1816–32. doi: 10.1091/mbc.e08-09-0894 19158374 PMC2655259

[26] Acevedo-Díaz A Morales-Cabán BM Zayas-Santiago A Martínez-Montemayor MM Suárez-Arroyo IJ . SCAMP3 regulates EGFR and promotes proliferation and migration of triple-negative breast cancer cells through the modulation of AKT, ERK, and STAT3 signaling pathways. Cancers (Basel). (2022) 14. doi: 10.3390/cancers14112807 35681787 PMC9179572

[27] Ali A Shafarin J Muhammad JS Farhat NM Hamad M Soofi A . SCAMP3 promotes breast cancer progression through the c-MYC-β-catenin-SQSTM1 growth and stemness axis. Cell Signal. (2023) 104:110591. doi: 10.1016/j.cellsig.2023.110591 36627007

[28] Li C Zhang Z Lv P Zhan Y Zhong Q . SCAMP3 promotes glioma proliferation and indicates unfavorable prognosis via multiple pathways. Onco Targets Ther. (2020) 13:3677–87. doi: 10.2147/ott.S242462 32431518 PMC7200257

[29] Morales-Cabán BM Cantres-Rosario YM Tosado-Rodríguez EL Roche-Lima A Meléndez LM Boukli NM . SCAMP3-driven regulation of ERK1/2 and autophagy phosphoproteomics signatures in triple-negative breast cancer. Int J Mol Sci. (2025) 26. doi: 10.3390/ijms26199577 41096842 PMC12525412

[30] Suárez-Arroyo IJ Feliz-Mosquea YR Pérez-Laspiur J Arju R Giashuddin S Maldonado-Martínez G . The proteome signature of the inflammatory breast cancer plasma membrane identifies novel molecular markers of disease. Am J Cancer Res. (2016) 6:1720–40. PMC5004075 27648361

[31] Tseng HW Li SC Tsai KW . Metformin treatment suppresses melanoma cell growth and motility through modulation of microRNA expression. Cancers (Basel). (2019) 11. doi: 10.3390/cancers11020209 30754729 PMC6406940

[32] Zhang X Sheng J Zhang Y Tian Y Zhu J Luo N . Overexpression of SCAMP3 is an indicator of poor prognosis in hepatocellular carcinoma. Oncotarget. (2017) 8:109247–57. doi: 10.18632/oncotarget.22665 29312605 PMC5752518

[33] Zhou A Liu H Tang B . Comprehensive evaluation of endocytosis-associated protein SCAMP3 in hepatocellular carcinoma. Pharmgenomics Pers Med. (2020) 13:415–26. doi: 10.2147/pgpm.S270062 33116758 PMC7548866

[34] Venugopalan A Lynberg M Cultraro CM Nguyen KDP Zhang X Waris M . SCAMP3 is a mutant EGFR phosphorylation target and a tumor suppressor in lung adenocarcinoma. Oncogene. (2021) 40:3331–46. doi: 10.1038/s41388-021-01764-y 33850265 PMC8514158

[35] Moschos SJ Sullivan RJ Hwu WJ Ramanathan RK Adjei AA Fong PC . Development of MK-8353, an orally administered ERK1/2 inhibitor, in patients with advanced solid tumors. JCI Insight. (2018) 3. doi: 10.1172/jci.insight.92352 29467321 PMC5916243

[36] Lakhani NJ Burris H Miller WH Jr. Huang M Chen LC Siu L . A phase 1b study of the ERK inhibitor MK-8353 plus pembrolizumab in patients with advanced solid tumors. Invest New Drugs. (2024) 42:581–9. doi: 10.1007/s10637-024-01461-z 39276176 PMC11625062

[37] Stathis A Tolcher AW Wang JS Renouf DJ Chen LC Suttner LH . Results of an open-label phase 1b study of the ERK inhibitor MK-8353 plus the MEK inhibitor selumetinib in patients with advanced or metastatic solid tumors. Invest New Drugs. (2023) 41:380–90. doi: 10.1007/s10637-022-01326-3 37040046 PMC10289957

[38] Wu TT Castle JD . Tyrosine phosphorylation of selected secretory carrier membrane proteins, SCAMP1 and SCAMP3, and association with the EGF receptor. Mol Biol Cell. (1998) 9:1661–74. doi: 10.1091/mbc.9.7.1661 9658162 PMC25404

[39] Wu TT Castle JD . Evidence for colocalization and interaction between 37 and 39 kDa isoforms of secretory carrier membrane proteins (SCAMPs). J Cell Sci. (1997) 110:1533–41. doi: 10.1242/jcs.110.13.1533 9224770

[40] Martinez-Osorio V Abdelwahab Y Ros U . The many faces of MLKL, the executor of necroptosis. Int J Mol Sci. (2023) 24. doi: 10.3390/ijms241210108 37373257 PMC10298923

[41] Maik-Rachline G Hacohen-Lev-Ran A Seger R . Nuclear ERK: mechanism of translocation, substrates, and role in cancer. Int J Mol Sci. (2019) 20. doi: 10.3390/ijms20051194 30857244 PMC6429060

[42] Huang WL Hsieh PF Chen SH Wu RC Mai HC Wu CH . SCAMP3 and EPS8 cooperatively regulate EGFR signaling to promote enzalutamide resistance and metastatic potential in prostate cancer. Cancer Genomics Proteomics. (2025) 22:953–70. doi: 10.21873/cgp.20549 41151858 PMC12577594

[43] Guo H Zhou C Zheng M Zhang J Wu H He Q . Insights into the role of derailed endocytic trafficking pathway in cancer: from the perspective of cancer hallmarks. Pharmacol Res. (2024) 201:107084. doi: 10.1016/j.phrs.2024.107084 38295915

[44] Naudin C Chevalier C Roche S . The role of small adaptor proteins in the control of oncogenic signalingr driven by tyrosine kinases in human cancer. Oncotarget. (2016) 7:11033–55. doi: 10.18632/oncotarget.6929 26788993 PMC4905456

[45] Lacombe L Hovington H Brisson H Mehdi S Beillevaire D Émond JP . UGT2B28 accelerates prostate cancer progression through stabilization of the endocytic adaptor protein HIP1 regulating AR and EGFR pathways. Cancer Lett. (2023) 553:215994. doi: 10.1016/j.canlet.2022.215994 36343786

[46] Pinilla-Macua I Sorkin A . Cbl and Cbl-b independently regulate EGFR through distinct receptor interaction modes. Mol Biol Cell. (2023) 34:ar134. doi: 10.1091/mbc.E23-02-0058 37903221 PMC10848940

[47] Young A Lou D McCormick F . Oncogenic and wild-type Ras play divergent roles in the regulation of mitogen-activated protein kinase signaling. Cancer Discov. (2013) 3:112–23. doi: 10.1158/2159-8290.Cd-12-0231 23103856

[48] Fortian A Sorkin A . Live-cell fluorescence imaging reveals high stoichiometry of Grb2 binding to the EGF receptor sustained during endocytosis. J Cell Sci. (2014) 127:432–44. doi: 10.1242/jcs.137786 24259669 PMC3889400

[49] Roux PP Blenis J . ERK and p38 MAPK-activated protein kinases: a family of protein kinases with diverse biological functions. Microbiol Mol Biol Rev. (2004) 68:320–44. doi: 10.1128/mmbr.68.2.320-344.2004 15187187 PMC419926

[50] Li D Miermont AM Sable R Quadri HS Mathews Griner LA Martin SE . Scaffolding protein connector enhancer of kinase suppressor of Ras 1 (CNKSR1) regulates MAPK inhibition responsiveness in pancreas cancer via crosstalk with AKT signaling. Mol Cancer Res. (2023) 21:316–31. doi: 10.1158/1541-7786.Mcr-21-1036 36790955 PMC10068447

[51] Kosako H Yamaguchi N Aranami C Ushiyama M Kose S Imamoto N . Phosphoproteomics reveals new ERK MAP kinase targets and links ERK to nucleoporin-mediated nuclear transport. Nat Struct Mol Biol. (2009) 16:1026–35. doi: 10.1038/nsmb.1656 19767751

[52] Villanueva J Yung Y Walker JL Assoian RK . ERK activity and G1 phase progression: identifying dispensable versus essential activities and primary versus secondary targets. Mol Biol Cell. (2007) 18:1457–63. doi: 10.1091/mbc.e06-10-0908 17314399 PMC1838994

[53] Aikin TJ Peterson AF Pokrass MJ Clark HR Regot S . MAPK activity dynamics regulate non-cell autonomous effects of oncogene expression. Elife. (2020) 9. doi: 10.7554/eLife.60541 32940599 PMC7498266

[54] Guerrero Zuniga A Aikin TJ McKenney C Lendner Y Phung A Hook PW . Sustained ERK signaling promotes G2 cell cycle exit and primes cells for whole-genome duplication. Dev Cell. (2024) 59:1724–1736.e4. doi: 10.1016/j.devcel.2024.03.032 38640927 PMC11233237

[55] Valenti M Molina M Cid VJ . Human gasdermin D and MLKL disrupt mitochondria, endocytic traffic and TORC1 signalling in budding yeast. Open Biol. (2023) 13:220366. doi: 10.1098/rsob.220366 37220793 PMC10205182

[56] Diegmiller R Salphati L Alicke B Wilson TR Stout TJ Hafner M . Growth-rate model predicts *in vivo* tumor response from *in vitro* data. CPT Pharmacometrics Syst Pharmacol. (2022) 11:1183–93. doi: 10.1002/psp4.12836 35731938 PMC9469692

[57] Finger AM Hendley AM Figueroa D Gonzalez H Weaver VM . Tissue mechanics in tumor heterogeneity and aggression. Trends Cancer. (2025) 11:806–24. doi: 10.1016/j.trecan.2025.04.004 40307158 PMC12350075

[58] Song D Lian Y Zhang L . The potential of activator protein 1 (AP-1) in cancer targeted therapy. Front Immunol. (2023) 14:1224892. doi: 10.3389/fimmu.2023.1224892 37483616 PMC10361657

