# Supplementary material for: Secretory carrier membrane protein 3 modulates nuclear ERK1/2 signaling and drug response in triple-negative breast cancer
Source: Front Oncol. 2026 Sep 14;16:1854966. doi: 10.3389/fonc.2026.1854966 (PMC13616617; doi:10.3389/fonc.2026.1854966)
Supplement: Supplementary file 1 [file SupplementaryFile1.zip › Supplementary_Material/Figure captions Supplementary Material - for resubmission -1.pdf]

Figure S1: Quantification of MAPK pathway protein expression in response to EGF and MK-8353 in TNBC cells.
(A, B) Densitometric quantification of total MAPK pathway protein levels in SUM-149 and MDA-MB-468 wild-type (WT) and SCAMP3 knockout (SC3KO) from immunoblots is shown in Figure 4. Data were analyzed using two-way ANOVA with Tukey´s multiple comparison test and are reported as mean ± SEM (n=3). $^{*}P \leq 0.05$, $^{**}P < 0.01$, $^{***}P < 0.001$, $^{****}P < 0.0001$ vs. NT within the same genotype; (#) indicates significance for WT vs. SC3KO under the indicated condition; (▪) indicates significance for EGF vs. MK-8353.

Figure S2: Protein expression of Importin-7.
Immunoblot and densitometric quantification of importin-7 in SUM-149 wild-type (WT) and SCAMP3 knockout (SC3KO) cells under basal conditions (NT), after EGF stimulation (10 ng/mL, 30 min), or following treatment with MK-8353 (8 µM, 2 h). Data were analyzed using two-way ANOVA with Tukey´s multiple comparison test and are reported as mean ± SEM (n=3). $^{*}P \leq 0.05$.

Figure S3: Quantification of MAPK pathway components in tumors.
(A) Densitometric quantification of total MAPK pathway protein levels in SUM-149 tumor lysates from vehicle- and MK-8353-treated mice from immunoblots showed in Figure 6. Data were analyzed using two-way ANOVA with Tukey´s multiple comparison test and are reported as mean ± SEM (n=5/group). $^{**}P < 0.01$, $^{***}P < 0.001$ vs. vehicle within each genotype; $^{\#\#}P < 0.01$, $^{\#\#\#}P < 0.001$ for WT vs. SC3KO under the indicated condition.

Figure S4: *In vivo* study with WT and SC3KO MDA-MB-468 xenografts.
MDA-MB-468 wild-type (WT) and SCAMP3 knockout (SC3KO) cells were orthotopically implanted into SCID mice, and tumor growth was monitored for nine weeks. **(A)** Mouse body weight over time. **(B)** Tumor growth curves. **(C)** Endpoint tumor weights. **(D)** Immunoblots of indicated proteins in tumor lysates. **(E)** Densitometric quantification. India ink was used as a loading control. The data were analyzed using an unpaired t-test and are reported as mean ± SEM (n = 4/group). $^{*}P \leq 0.05$; ns = not significant.
