# Supplementary material for: Secretory carrier membrane protein 3 modulates nuclear ERK1/2 signaling and drug response in triple-negative breast cancer
Source: Front Oncol. 2026 Sep 14;16:1854966. doi: 10.3389/fonc.2026.1854966 (PMC13616617; doi:10.3389/fonc.2026.1854966)
Supplement: Supplementary file 1 [file SupplementaryFile1.zip › Supplementary_Material/Figure_S1.pdf]

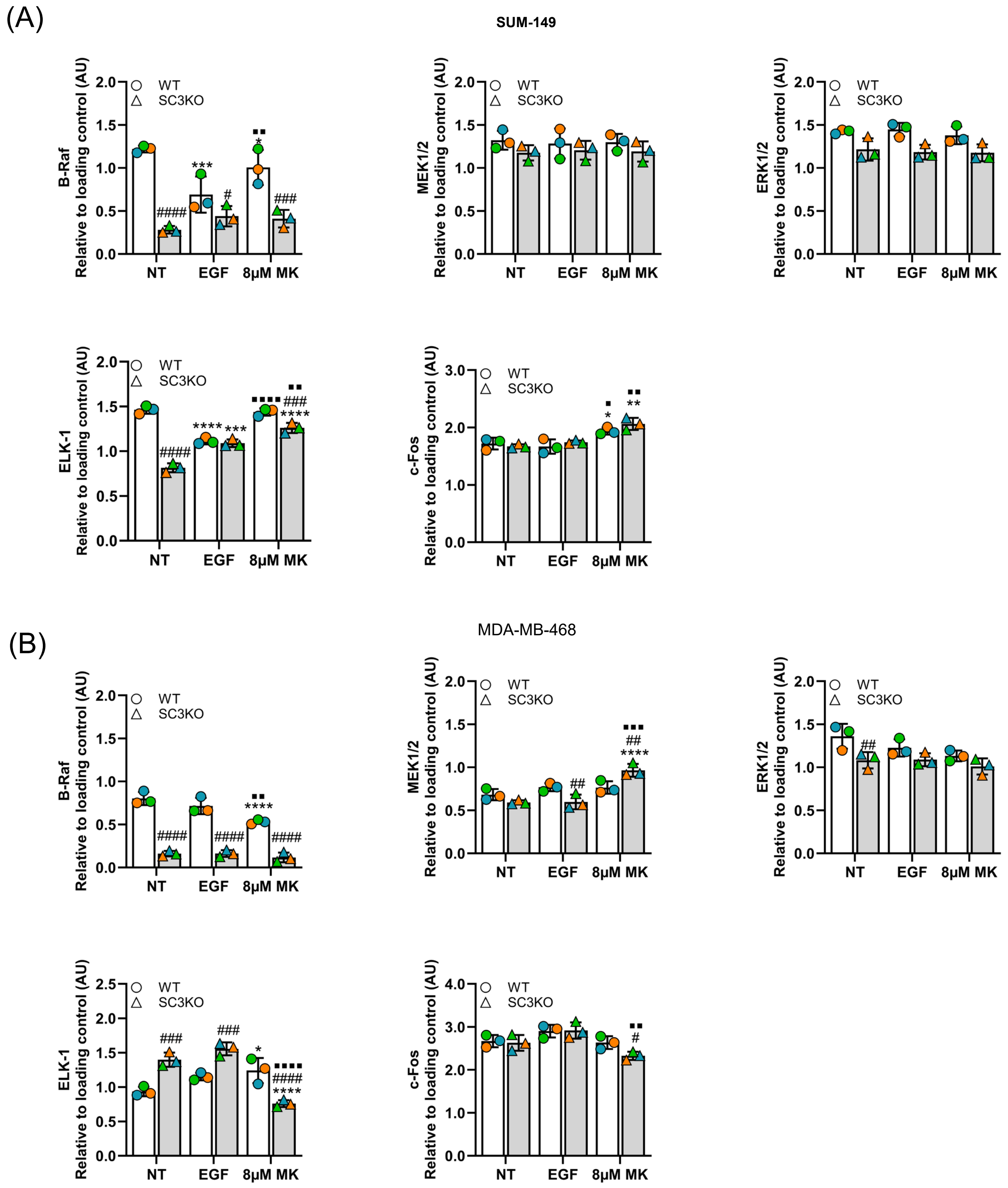

(A)
SUM-149
B-Raf
MEK1/2
ERK1/2
ELK-1
c-Fos
Relative to loading control (AU)
WT
SC3KO
NT
EGF
8µM MK
####
***
#
*
###
****
**
(B)
MDA-MB-468
##
