# Supplementary material for: Secretory carrier membrane protein 3 modulates nuclear ERK1/2 signaling and drug response in triple-negative breast cancer
Source: Front Oncol. 2026 Sep 14;16:1854966. doi: 10.3389/fonc.2026.1854966 (PMC13616617; doi:10.3389/fonc.2026.1854966)
Supplement: Supplementary file 1 [file SupplementaryFile1.zip › Supplementary_Material/Figure_S2.pdf]

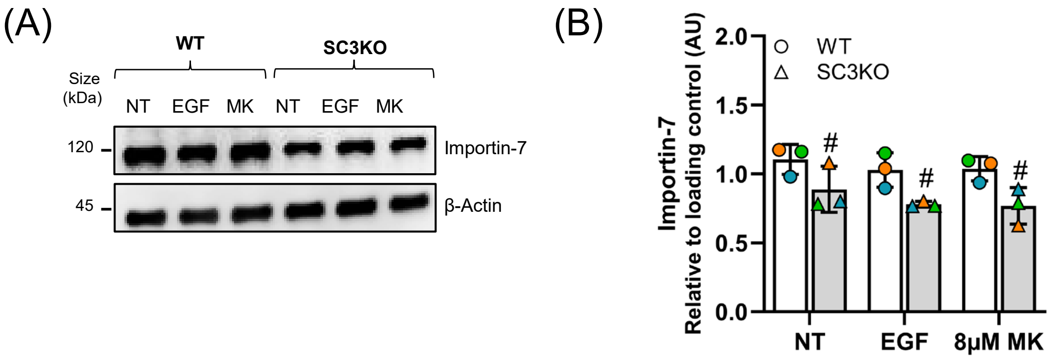
(A)
WT
SC3KO
Size
(kDa)
NT
EGF
MK
NT
EGF
MK
120
45
Importin-7
β-Actin
(B)
Importin-7
Relative to loading control (AU)
2.0
1.5
1.0
0.5
0.0
WT
SC3KO
#
#
#
NT
EGF
8μM MK
