# Supplementary material for: Secretory carrier membrane protein 3 modulates nuclear ERK1/2 signaling and drug response in triple-negative breast cancer
Source: Front Oncol. 2026 Sep 14;16:1854966. doi: 10.3389/fonc.2026.1854966 (PMC13616617; doi:10.3389/fonc.2026.1854966)
Supplement: Supplementary file 1 [file SupplementaryFile1.zip › Supplementary_Material/Figure_S3.pdf]

(A)

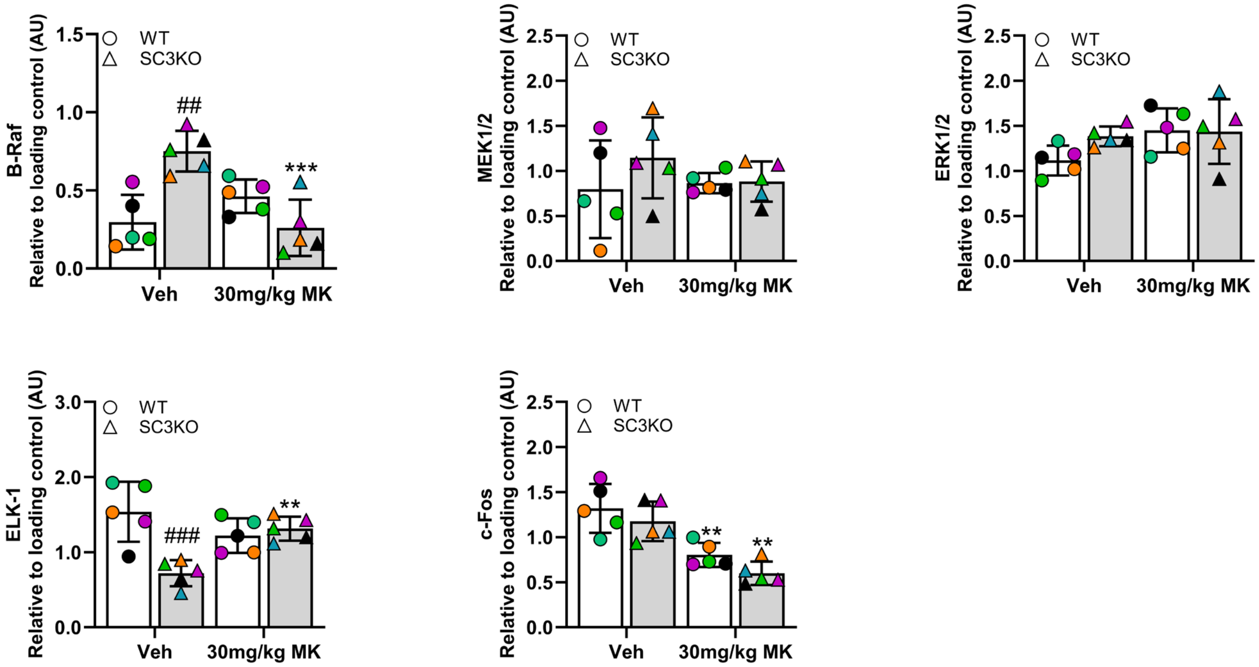

B-Raf
Relative to loading control (AU)
1.5
1.0
0.5
0.0
WT
SC3KO
##
***
Veh
30mg/kg MK
MEK1/2
Relative to loading control (AU)
2.5
2.0
1.5
1.0
0.5
0.0
WT
SC3KO
Veh
30mg/kg MK
ERK1/2
Relative to loading control (AU)
2.5
2.0
1.5
1.0
0.5
0.0
WT
SC3KO
Veh
30mg/kg MK
ELK-1
Relative to loading control (AU)
3.0
2.0
1.0
0.0
WT
SC3KO
###
**
Veh
30mg/kg MK
c-Fos
Relative to loading control (AU)
2.5
2.0
1.5
1.0
0.5
0.0
WT
SC3KO
**
**
Veh
30mg/kg MK
