# Supplementary material for: Secretory carrier membrane protein 3 modulates nuclear ERK1/2 signaling and drug response in triple-negative breast cancer
Source: Front Oncol. 2026 Sep 14;16:1854966. doi: 10.3389/fonc.2026.1854966 (PMC13616617; doi:10.3389/fonc.2026.1854966)
Supplement: Supplementary file 1 [file SupplementaryFile1.zip › Supplementary_Material/Figure_S4.pdf]

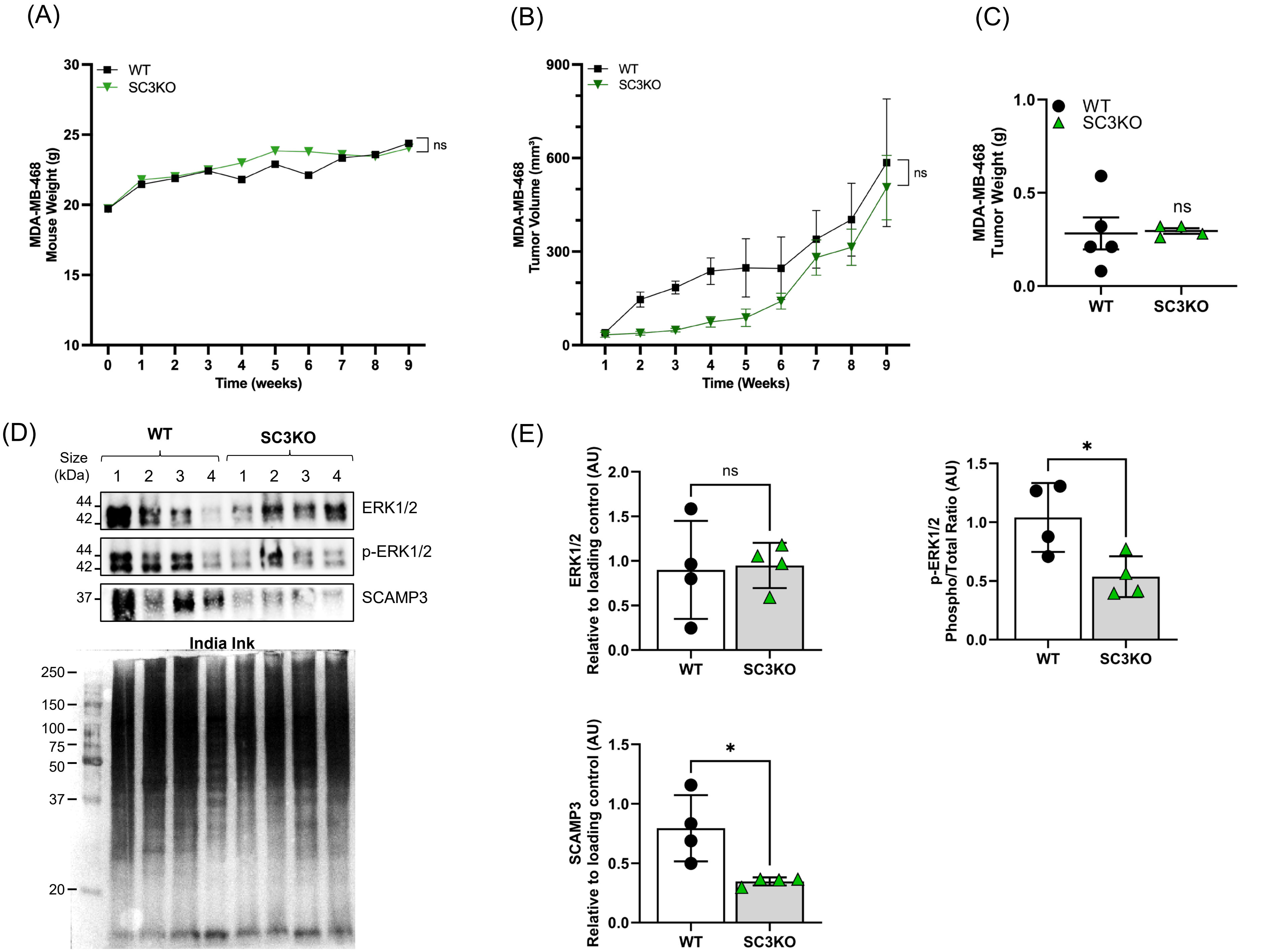

(A)
WT
SC3KO
ns
MDA-MB-468
Mouse Weight (g)
Time (weeks)
(B)
WT
SC3KO
ns
MDA-MB-468
Tumor Volume (mm³)
Time (Weeks)
(C)
WT
SC3KO
ns
MDA-MB-468
Tumor Weight (g)
(D)
WT
SC3KO
Size (kDa)
ERK1/2
p-ERK1/2
SCAMP3
India Ink
(E)
ERK1/2
Relative to loading control (AU)
ns
p-ERK1/2
Phospho/Total Ratio (AU)
*
SCAMP3
Relative to loading control (AU)
*
